# Structure–function coupling using fixel-based analysis and functional magnetic resonance imaging in Alzheimer’s disease and mild cognitive impairment^1^

**DOI:** 10.1162/netn_a_00461

**Published:** 2025-07-29

**Authors:** Charly Hugo Alexandre Billaud, Junhong Yu, for the Alzheimer’s Disease Neuroimaging Initiative

**Affiliations:** Nanyang Technological University, Psychology, School of Social Sciences, 48 Nanyang Avenue, Singapore

**Keywords:** Connectivity, Multimodal, fMRI, DWI, Fixel-based analysis, Aging

## Abstract

Functional MRI (fMRI) and diffusion-weighted imaging (DWI) help explore correlations between structural connectivity (SC) and functional connectivity (FC; SC–FC coupling). Studies on mild cognitive impairment (MCI) and Alzheimer’s disease (AD) observed coupling disruptions, co-occurring with cognitive decline. Advanced “fixel-based” analyses improved DWI’s accuracy in assessing microstructural and macrostructural features of white matter (WM), but previous aging coupling studies commonly defined SC via tensor-based tractography and streamline counts, thereby missing fiber-specific information. We investigated different types of fixel-FC coupling and their relation to cognition in 392 participants (Age_mean_ = 73; 207 females) from the ADNI. Two hundred twenty-five controls, 142 MCI, and 25 AD with diffusion-weighted and resting-state fMRI scans were analyzed. Structural connectomes were constructed using average fixel metrics (fiber density (FD), fiber-bundle cross-section log, and combined [FDC]) as edges. SC–FC coupling for each SC metric was calculated at overall network, edge, and node levels. Overall DMN, node- and edge-specific coupling differences were found across SC measures and groups. DMN nodal coupling significantly predicted Mini-Mental Status Examination score and verbal memory. In conclusion, different types of fixel-based coupling alterations can be observed across the neurocognitive aging spectrum, in particular, FD–FC and FDC–FC coupling between DMN regions are associated with cognitive functioning.

## Author Summary

Structure–function coupling is the correlation between white matter structures connecting pairs of brain regions (structural connectivity) and their concomitant brain activity (functional connectivity). “fixel-based” diffusion-weighted imaging techniques improve the accuracy of white matter structure estimation and provide information on microstructural and macrostructural changes to brain networks. Using structural connectomes based on fixel-based weights, coupling was found to be altered in Alzheimer’s disease and mild cognitive impairment, especially in the default mode network. Default mode network coupling may be relevant to verbal memory and cognitive decline across aging, particularly for coupling driven by microstructural features of white matter.

## INTRODUCTION

### Aging and the Default Mode Network

Recording an individual’s brain activity during a conscious “resting state” (or “task-free” recording) is a common way to explore brain networks using functional magnetic resonance imaging (fMRI). fMRI captures the blood-oxygenation-level–dependent (BOLD) signal that accompanies activating populations of neurons. It allows researchers to estimate a “functional connectivity” (FC) between pairs of brain regions when their BOLD activation is statistically associated across time (Fox & Raichle, 2007). Resting-state fMRI brain recordings helped identify brain areas that activate together when an individual is awake and at rest; one example is the “default mode” network (DMN) of regions (Fox & Raichle, 2007; Raichle et al., 2001; Smallwood et al., 2021). Activity within the DMN has been associated with a range of cognitive functions including working memory, semantic memory, rule-based sorting, and social cognition (Menon, 2023; Smallwood et al., 2021). Longitudinal reductions in DMN resting-state functional connectivity (rsFC) in a healthy aging cohort were also found in the precuneus and posterior cingulate cortex (pCunPCC; Malagurski et al., 2022), which overlaps with DMN changes in mild cognitive impairment (MCI) and Alzheimer’s disease (AD; Badhwar et al., 2017; Eyler et al., 2019). There may, thus, be a common evolution of DMN across the neurocognitive aging spectrum.

Clinically focused investigations highlighted increases and decreases in connectivity to DMN regions in aging-related neurological disorders including MCI (Y. Cao et al., 2021; Celebi et al., 2016; Eyler et al., 2019; Malotaux et al., 2023; Zhou et al., 2022) and AD (Badhwar et al., 2017; Dai et al., 2019; Jones et al., 2016; Mohan et al., 2016; Zhou et al., 2022), affecting the medial temporal lobe (MTL), medial prefrontal cortex (mPFC), anterior cingulate cortex, and most commonly, the pCunPCC (Badhwar et al., 2017; Eyler et al., 2019; C. Wang et al., 2018; Y. Wang et al., 2024). DMN functional dysconnectivity also correlated with deficits in executive functions, attention, episodic memory, and verbal memory (Celebi et al., 2016; Weiler et al., 2014; H. Yang et al., 2017; Yao et al., 2021). Whether DMN “coupling,” the relationship between structural connectivity (SC) and FC, is associated with cognitive performance in those diagnosed with AD or MCI remains to be investigated.

### Structure–Function Coupling With Diffusion-Weighted Imaging (DWI)

SC, that is, neural connections estimated with white matter (WM) fibers, is also tied to the transmission of information across the brain network, and thus likely influencing FC. In past DWI studies, estimates of the integrity of WM, commonly using diffusion tensor imaging (DTI), have been related to fMRI FC in both healthy (Bennett & Rypma, 2013) and clinical populations including AD and MCI (R. Cao et al., 2020; Dai et al., 2019; Sun et al., 2014; J. Wang et al., 2018; Zhou et al., 2022). Correlations (or *coupling*) between rsFC and SC estimated from conventional tractography approaches have been reported in typical aging, MCI, and AD, with varying strength (R. Cao et al., 2020; Dai et al., 2019; Sun et al., 2014). Besides SC or FC on their own, structure–function coupling is therefore a distinct feature of brain network organization in aging. Multimodal coupling was thus the focus of this study instead of isolated SC or FC.

Only a few studies have attempted to investigate whether multimodal coupling was relevant to cognitive performance in neurocognitive aging and highlighted differences between cognitively normal (CN) older adults, AD, and MCI (R. Cao et al., 2020; Sun et al., 2014; J. Wang et al., 2018), with coupling correlating to clinical measures of cognitive decline such as the Mini-Mental State Examination (MMSE; R. Cao et al., 2020). This suggests that SC–FC coupling is relevant to neurocognitive aging.

### Structure–Function Coupling With Fixel-Based Analysis

Recent advances in modeling DWI data have enabled one to estimate crossing fiber populations (“fixels”) within the image voxels (Jeurissen et al., 2013; Mito et al., 2018). Because fixel-based analyses (FBAs) improve upon conventional DTI within-voxel averaging, they can identify WM tracts more accurately (Dhollander et al., 2021; Jeurissen et al., 2013; Raffelt et al., 2017). Furthermore, FBA can describe microstructural aspects of the SC by estimating apparent fiber density (FD; from the radial water diffusion, which is proportional to the intra-axonal volume [Raffelt et al., 2012]); and macrostructural aspects of the SC by estimating fiber-bundle cross-section (FbC); both summarized into a single global measure (fiber density and cross-section, FDC; Dhollander et al., 2021; Raffelt et al., 2017). This provides additional information on the nature of WM tracts that SC measures based on the number of streamlines (Smith et al., 2022; Sun et al., 2014; Zhou et al., 2022), or the probability of streamlines connecting regions (C. Wang et al., 2018), cannot give. However, at present, not much is known about these FBA metrics in relation to FC, and most studies investigating coupling in neurocognitive aging have relied on tensor-based tractography (R. Cao et al., 2020; Dai et al., 2019; Sun et al., 2014; Zhou et al., 2022).

Previous studies reporting fixel-wise alterations in AD and MCI hypothesized that there could be a parallel between altered functional networks known to these groups and altered WM tracts (Mito et al., 2018; F. P. G. Yang et al., 2021). This makes sense as fibers with more axons are expected to transmit more information, thereby leading to increasing activity between connected regions. One study in adults reported fixel–FC coupling in autism spectrum disorder (Dong et al., 2023). However, no study, to our knowledge, has attempted to investigate that relationship directly in neurocognitive aging. Given alterations in the DMN’s rsFC are associated with cognitive deficits (Celebi et al., 2016; Weiler et al., 2014; H. Yang et al., 2017; Yao et al., 2021), and that SC–FC coupling is tied to cognitive decline in AD and MCI (R. Cao et al., 2020), we speculate that fixel-wise SC–FC coupling in the DMN will likewise predict cognitive performance in this population.

We investigated the fixel-based SC–FC coupling alterations across the neurocognitive disorder spectrum. In line with previous coupling studies that used other SC methods, we hypothesized that:

- fixel-based metrics (FD, FbC, FDC) connectomes correlate positively with rsFC connectomes;
- fixel-based SC-FC coupling differs between CN controls, MCI, and AD populations; and
- DMN-specific edges and nodes that differ in coupling compared with controls predict cognitive performance.

## METHODS

### Participants

In the current study, we analyzed data from the Alzheimer’s Disease Neuroimaging Initiative (ADNI, adni.loni.usc.edu). The ADNI is a partnership started in 2003 under the direction of Michael W. Weiner, MD, to investigate the combination of serial MRI, positron emission tomography (PET), and other biological markers, as well as clinical and neuropsychological assessment to measure the progression of MCI and early AD (see www.adni-info.org for complete and up-to-date information). Details regarding the criteria for dementia and MCI used in the ADNI are available at https://adni.loni.usc.edu/wp-content/uploads/2008/07/adni2-procedures-manual.pdf. Ethical approval for the ADNI study was obtained by the ADNI investigators.

Participants from the ADNI-3 database were selected across the neurocognitive aging spectrum (CN with or without subjective memory complaints, and meeting diagnostic criteria for MCI and AD) to maximize variability in the cognitive and neuroimaging measures. From the ADNI-3 database, we included participants if they had a valid T1-weighted, resting-state fMRI and “ADNI-3 Basic” diffusion scans. Participants who were scanned only with the “ADNI-3 Advanced” diffusion protocol were not included due to incompatibility in the protocols.

The demographics of the included participants are reported in Table 1. Age did not significantly differ across groups, *F*(2, 389) = 2.263, *p* = 0.105, nor did years of education, *F*(1, 390) = 0.611, *p* = 0.435, as suggested by the results of one-way between-subjects analyses of variance (ANOVAs). Sex distribution did significantly differ across groups, *χ*^2^(2) = 16.131; *p* ≤ 0.001), with CN having significantly more females in proportion (adj. standard residual: 3.72).

**Table 1.** Demographic information of the dataset

Characteristics	Total *N* = (392)	CN *N* = (225)	MCI *N* = (142)	AD *N* = (25)
Age in years (*M* ± *SD*)	73.0 ± 8	72.3 ± 8	73.9 ± 8	74.7 ± 8
Sex M:F	185:207	88:137	79:63	18:7
Education years (*M* ± *SD*)	16.4 ± 2	16.7 ± 2	16.3 ± 3	15.6 ± 2

*Note*. CN = cognitively normal. MCI = mild cognitive impairment. AD = Alzheimer’s disease. M = male. F = female.

### Neuropsychological Assessment

The Rey Auditory Verbal Learning Test (RAVLT) completed is a validated measure of declarative memory (Van der Elst et al., 2005). In the RAVLT, each participant is given a list (List A) of 15 unrelated words to learn and recall immediately aloud over five trials (immediate recall test). Each is then given an interference list (List B) of 15 unrelated words once, to learn and immediately recall; after that, the participant is required to recall aloud List A’s words. After a 30-min delay, each is asked again to recall List A’s words (30-min delay recall). Finally, a 50-word list is given to each participant, including words from lists A and B, and 20 new distractor words, from which each had to identify words of List A (Recognition).

Only 349 out of 392 participants completed the RAVLT measures necessary to estimate the memory composite score (203 CN, 127 MCI, 19 AD). Only one participant (CN) did not complete the MMSE.

### Neuroimaging Acquisition

The neuroimaging acquisition protocol is summarized in Table 2. Alzheimer's Disease Neuroimaging Initiative - 3 (ADNI3) data varied across scanners and protocols (https://adni.loni.usc.edu/), with *b* = 1,000 s/mm^2^ volumes in 16 to 55 directions (*M* = 44.5), and 1 to 7 *b* = 0 s/mm^2^ values (*M* = 4.3) per DWI scan.

**Table 2.** Neuroimaging protocol

Sequence	Dimensions (mm)	Parameters	Time
T1 (accelerated sagittal MPRAGE)	208 × 240 × 256	TE = min full echo TR = 2,300 TI = 900	6:20
rsfMRI (EPI-BOLD)	220 × 220 × 163	TE = ~30; TR = 3,000; FA = 90°	10:00
Diffusion-weighted	232 × 232 × 160	TE = 56; TR = 7,200; single-shell b = 1,000s/mm^2^	7:30

*Note*. Refer to ADNI3 (https://adni.loni.usc.edu/) for complete information on MRI protocols: https://adni.loni.usc.edu/wp-content/themes/freshnews-dev-v2/documents/mri/ADNI3_MRI_Analysis_Manual_20180202.pdf. MPRAGE = magnetization prepared rapid gradient echo imaging. rsfMRI = resting-state functional magnetic resonance imaging. EPI = echo-planar imaging. BOLD = blood-oxygenation-level–dependent. TE = echo time. TR = repetition time.

### Resting-State fMRI Preprocessing

Resting-state fMRI scans were preprocessed using *fMRIPrep* 20.2.5 (Esteban et al., 2019). Slice time correction was applied to the functional data with the Analysis of Functional NeuroImages (AFNI) software’s *3dTshift* function (Cox, 1996), and motion correction using FSL’s MCFLIRT algorithm (Jenkinson et al., 2002). Each fMRI scan was coregistered to a respective T1-weighted image with the bbregister program (boundary-based registration) in FreeSurfer (Greve & Fischl, 2009), with 9 degrees of freedom. The antsApplyTransforms algorithm (Avants et al., 2009) and the Lanczos interpolation were used for motion correction, BOLD-to-T1w transformation and T1w-to-template (MNI) warp. The Python package Nilearn (https://github.com/nilearn) was used to denoise the volumes by regressing six motion translation/rotation parameters, the average WM signal, cerebrospinal fluid (CSF) masks, the global signal and derivatives, and discrete cosines covering the slow-time drift frequency band. Excessive motion was additionally removed via scrubbing (Power et al., 2014). The volumes were smoothed using a 5-mm full width at half maximum (FWHM) kernel and subjected to a 0.1-Hz low-pass filter. Participants were excluded if they had excessive head motion (>20% of their fMRI volumes with relative RMS [root-mean squared] displacement >0.25).

### Functional Connectome Construction

A 119 × 119 atlas in MNI space based on the parcellations of the seven-network Schaefer 100 atlas (Schaefer et al., 2018) and 19 subcortical parcellations from the *aseg* atlas (Fischl et al., 2002) was selected to build both the functional and structural connectomes. The functional connectome was built by defining each edge as the average BOLD Pearson *r* correlation, Fisher-*Z* transformed, between pairs of regions. Although negative functional connections are difficult to interpret, and their weight has been set to zero in past network coupling analyses (Dai et al., 2019), we have no a priori assumptions on the functional behavior at rest of the *existing* structural edges and whether fixel-weights are relevant or irrelevant to this phenomenon. Negative edges were therefore kept as such and left untouched. Supporting Informations S3 and S4 contain exploratory analyses with only positive or only negative FC edges separately.

### DWI Preprocessing

The diffusion-weighted images were preprocessed with MRTrix v3.0.4 (https://www.mrtrix.org/; Dhollander & Connelly, 2016; Raffelt et al., 2017; Tournier et al., 2019). Preprocessing of DWI data was facilitated by the use of GNU parallel, which allows parallel processing in multiple CPU threads (Tange, 2024). Marcenko-Pastur Principal Components Analysis (MP-PCA) denoising of the DWI images (Veraart et al., 2016) was applied with Gibbs-ringing artifact removal (Kellner et al., 2016), distortion correction, and N3 bias field correction (Tustison et al., 2010).

The Dhollander algorithm (Dhollander et al., 2019) was used to obtain the average response function of WM, gray matter (GM), and CSF from the corrected images, and the images were upsampled to 1.25 mm in isotropic voxel size. Multishell, multitissue constrained spherical deconvolution was used as it is applicable on single-shell data with *b* = 0 and *b* = 1,000 values; Jeurissen et al., 2014), fiber orientation distribution (FOD) was estimated from the WM and CSF response functions, and normalized via the *mtnormalise* command. Following the approach of two previous studies (Luo et al., 2021; Mito et al., 2018), a study-specific FOD template was generated from a representative subsample of 40 random participants including 10 CN, 10 MCI, 10 subjective memory complaint (SMC), and 10 AD; each group with five females and five males, and a similar age distribution as the whole sample (Kolmogorov–Smirnov at *p* > 0.05). The FOD of each participant was registered to the template, and a template whole brain mask was created to define the fixels of interest. The cerebellum was manually excluded using the freeview tool in FreeSurfer (Fischl, 2012). A template fixel mask was defined using connectivity-based fixel enhancement (Raffelt et al., 2015) and manually visualized to ensure the inclusion of fixels within WM and not within GM (−fmls_peak_value was optimally set at 0.15 in this dataset), resulting in a mask of 291,685 fixels.

Individual fixel data were matched to the template fixel mask to obtain a fixel–fixel correspondence. The apparent FD, log of fiber-bundle cross-section (log(FbC)) and the FDC measures were obtained in each participant’s fixel data (Dhollander et al., 2021). A whole-brain fiber tractogram with 20 million streamlines was created based on the FOD template (amplitude cutoff: 0.15) with MRtrix3’s iFOD2 algorithm and downsampled to 2 million fibers using the spherical-deconvolution informed filtering of tractograms (SIFT) algorithm (Smith et al., 2013). The tractogram was used to estimate fixel–fixel connectivity for each measure and then smooth the fixel values across connected fixel neighbors (fixelfilter, at 10 mm).

### Fixel-Based Connectome Construction

To align the same 119-parcel atlas with the FOD template’s space, the Advanced Normalization Tools (Tustison et al., 2021) were first used to register the MNI152 (1 mm T1) template to the FOD template. After generating an identity warp (deformation field) from the registered template with MRTrix, and transforming it using antsApplyTransforms, both the MNI template and the 119-label atlas based on the former were warped to the FOD template with nearest interpolation using the mrtransform command. This allowed the 119 parcellations to be aligned with the fixel data.

**Figure 1.**
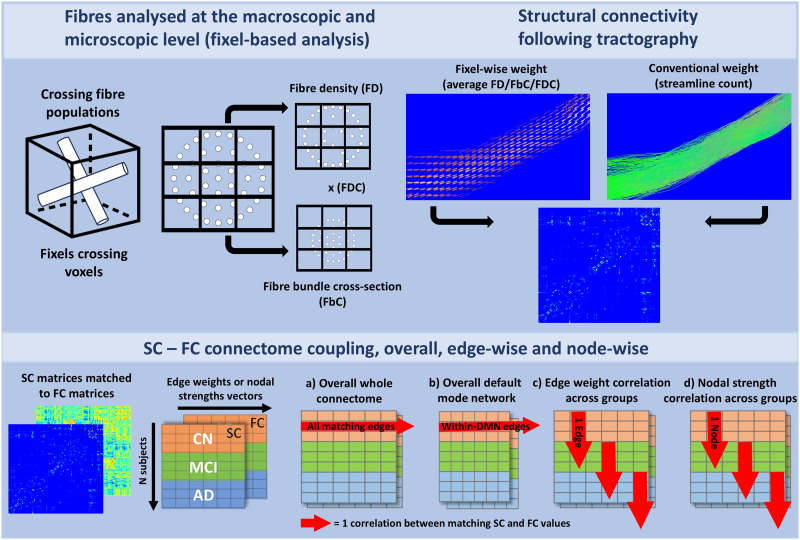
Structural connectivity–functional connectivity coupling protocol. The upper panel describes “fixels” (white cylinders/circles), which are estimations of fiber populations crossing within a given voxel (black box/grid squares). FD estimates changes in within-voxel fiber density, and FbC estimates the macroscopic change in the fiber bundle’s cross-section. Fixel-wise SC measures averaged fiber-specific estimates along a WM tract, while conventional SC was defined as the number of streamlines generated along the tract via probabilistic tractography. The bottom panel shows how SC–FC coupling was estimated from vectors of FC connections (weighted BOLD signal correlation) and their corresponding SC connections (weighted either with average fixel-wise metrics or streamline count), built from the matrices. The matching vectors were correlated together at subject level (horizontal red arrow) for all edges of the whole connectome (a) or edges within the default mode network (b); as well at group level (vertical red arrow) for coupling of specific edge vectors (c) or nodal strength vectors (d).

Using the whole-brain tractogram and the warped atlas, the streamlines that connected each pair of brain regions were estimated and individually extracted to create a fixel mask for each structural connection. The edge streamline assignment was based on the region-of-interest (ROI)’s voxels reached at both *ends* of each streamline, regardless of ROIs that potentially intersected along the streamline’s length (default tck2connectome function parameters). 5,922 edges in the 119 × 119 matrix were assigned at least one streamline. Each fixel mask was used to compute the average FD, average log(FbC) and average FDC values of the fixels (Figure 1, top panel) within the masks for all participants, ignoring zero values. These average values were selected to define the edge weights of subjects’ 119 × 119 SC matrices. To avoid giving the same weight to edges with different numbers of streamlines (but the same average fixel-wise value), each average FD/FbC/FDC metric was multiplied by the number of streamlines used to define the respective edge. Missing edges were set to not available (NA).

### Conventional Streamline Connectome Construction

Within MRtrix3, the FSL five-tissue-type segmentation of each T1-weighted image generates a mask. Anatomically constrained tractography was applied on this mask, allowing anatomically realistic streamlines to be estimated from the FOD (Smith et al., 2012). Here, each individual’s normalized WM FOD was used to estimate 2,000,000 streamlines per participant (default cutoff, no SIFT filtering applied: on average, 11,676 edges (*SD* = 1,093) were assigned at least one streamline). From individual tractography, SC matrices were generated for each subject using the same 119-parcel atlas, registered to the individual DWI, with edges being weighted as the number of streamlines. Streamline-count-based SC is referred here as “streamline SC.” Missing edges (0 streamline assignment) were set to NA.

### Statistics

SC–FC coupling was investigated in multiple modalities and at specific levels of the connectome, which is summarized in Figure 1. Code for the coupling analyses is available at https://github.com/chabld/FBA_coupling. As depicted in the bottom panel, each participant’s SC and FC adjacency connectivity matrices were converted so that each participant had two vectors of edge weights (corresponding to all the atlas’ pairs of regions, without the diagonal of self-connected regions and reverse directions), one for SC and one for FC. For nodal analyses, each participant also had two vectors of 119 nodes and their “strength” value for SC and FC, which we describe further down the section. Following previous coupling protocols in this population (R. Cao et al., 2020; Sun et al., 2014), only direct connections were kept to measure SC or FC weights (i.e., connectivity between two regions did not account for intermediate or relay regions in the connectome).

Statistical analyses were carried out in *R* v.4.3.2 (R Core Team, 2023). Whole-connectome overall SC–FC coupling (Figure 1A) was computed by correlating all SC edges and matching FC edges first in each participant connectome. The effect of group on participant-wise overall coupling was tested using an ANOVA model controlling for age and sex. If the ANOVA showed a significant between-group effect, post hoc comparisons were investigated with estimates of means or marginal means via the emmeans R package v.1.10.6-0900030 (Lenth, 2025). Overall coupling was additionally tested on the group-averaged SC and FC to replicate the approach of previous studies (Sun et al., 2014; J. Wang et al., 2018): each SC edge and FC edge value was averaged across participants of each group separately, and then correlated (giving one Pearson *r* value per group). The resulting group-wise *r*s were compared across groups with Fisher’s *r*-to-*Z* transform in the *cocor* package (Diedenhofen & Musch, 2015; Fisher, 1925). Following Dai and colleagues (2019), the same overall analyses were run only for within-DMN edges (Figure 1B). Exploratory comparisons for other networks of the Yeo-7 network and of the aseg subcortical ROIs are appended in Supporting Information S1.

To investigate edge-specific coupling (Figure 1C), individual SC values were correlated to matching FC values of each edge across participants per group separately, resulting in one coupling *r* value per edge for each group. Participants had one SC and one FC value for a given edge, so the correlation was between SC and FC from all participants of a group. For each edge, the difference in coupling between groups was calculated by subtracting one group’s edge-wise *r* value from another group’s edge-wise *r* value. Statistical significance was set with a two-tailed threshold at *p* < 0.01 (with Bonferroni correction across the four SC metrics), determined from the distribution of differences obtained with between-group shuffling of the SC and FC edge values before their coupling/correlation (5,000 permutations).

To investigate node-specific coupling (Figure 1D), individual SC nodal-strength values were correlated to matching FC nodal strength values, across participants of each group separately, resulting in one coupling *r* value per node for each group. Nodal strength was defined as the sum of the weights of edges connected to the node, using the *igraph* package v.2.0.3 in R (Csardi & Nepusz, 2006) with the default arguments in the strength() function. Each of the 119 nodes was assigned one SC and one FC strength value (provided connections to that node existed) for every participant. To get the most accurate strength of each node, FC strength was not limited to functional connections that also had an existing SC weight. Coupling was calculated by correlating matching SC and FC nodal strength values, across participants of each group separately. Like in the edge-wise analysis, the difference in coupling between groups was calculated by subtracting one group’s node-wise coupling value from another group’s node-wise *r* value. Statistical significance was set with a two-tailed threshold at *p* < 0.01 (with Bonferroni correction across the four SC metrics), determined from the distribution of differences obtained with between-group shuffling of the SC and FC node strength values before their coupling/correlation (5,000 permutations).

To assess the effect of node-coupling and edge-coupling on cognition, SC and FC vectors of DMN node strengths and within-DMN edge weights were selected in each participant. This was done at the participant level, with a coupling value covering all edges or nodes of the DMN in a participant’s SC/FC vector, not edge-specific or node-specific with one value per group as in the previous two comparison analyses. Default-mode network edges and nodes were categorized for specific statistical analyses based on the “Default” network label classification from the seven-network Schaefer 100 atlas (Yeo et al., 2011), which includes 24 nodes. The couplings of DMN edges and DMN nodes were calculated in each participant by correlating two matching SC and FC vectors of DMN edge weights, and correlating two matching SC and FC vectors of node strengths. This resulted in two coupling values, one for DMN nodes and one for DMN edges, in each participant. For each SC metric separately, a linear regression model included the two DMN coupling measures (i.e., edges and nodes), controlling for age, sex, and estimated total intracranial volume (computed with *FreeSurfer*). Multicollinearity was assessed in each model by assessing if its variance inflator index exceeded 10 (Mayers, 2013; Myers, 1990), and tolerance statistics were less than 0.02 (Mayers, 2013; Menard, 2001). We also checked for any intercorrelations between predictors that exceeded 0.8. Each model was repeated twice, one with MMSE as the dependent variable, and the second with verbal memory as the dependent variable. Verbal memory was transformed into one global measure using the Learning, Immediate, 30-min delay recall, and Recognition scores from the RAVLT, a latent factor composite memory score (scaled from 0 to 1) was estimated in a confirmatory factor analysis model according to a previous study (Billaud, Yu, & Alzheimer’s Disease Neuroimaging Initiative, 2024), using the *lavaan* package (Rosseel, 2012).

## RESULTS

### Overall Whole-Connectome and DMN Coupling

**Figure 2.**
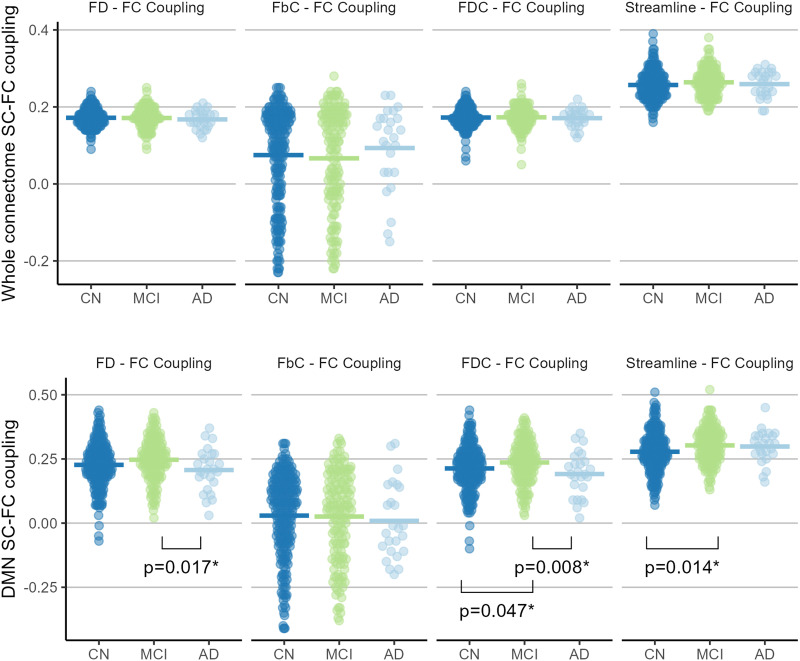
Subject-wise overall coupling for the whole connectome and the default mode network across groups and structural connectivity metrics. Each dot represents a participant’s Pearson *r* correlation between the weights of FC and SC edges for the whole connectome (top panel) and for within-DMN edges (bottom panel). The colored thick bars represent the group mean *r*. *Comparisons significant at *p* < 0.05 (do not survive correction for false discovery rate), obtained with post hoc contrast of estimates of marginal means.

The subject-wise overall coupling, for edges of the whole connectome and of the DMN, across diagnosis groups and SC metrics, are depicted in Figure 2. Diagnosis group had no significant effect on subject-wise whole-connectome coupling for any fixel-based measure (FD: *F* = 0.334, *p* = 0.716; log(FbC): *F* = 0.585, *p* = 0.557; FDC: *F* = 0.078, *p* = 0.925, respectively), nor for streamline SC (*F* = 1.656, *p* = 0.192). Diagnosis group had no significant effect on subject-wise overall DMN coupling for log(FbC)–FC coupling (*F* = 0.194, *p* = 0.824) but did for coupling with the other SC measures (FD: *F* = 3.739, *p* = 0.0246; FDC: *F* = 4.806, *p* = 0.0087, streamline SC: *F* = 5.189, *p* = 0.006). Post hoc tests, depicted in Figure 2, revealed group differences between AD < MCI for FD–FC, *t*(387) = −2.40, and FDC–FC coupling, *t*(387) = −2.66; and MCI and CN in streamline SC–FC coupling (CN < MCI, *t*(387) = −2.47). Differences were also present in the Yeo-7 Ventral Attention networks for streamline SC–FC coupling (Supporting Information S1).

The whole-connectome and within-DMN overall coupling for group averages are depicted in Table 3. No significant difference in coupling was detected between group averages for any SC measure, either for the whole connectome or for connections within the DMN (all *p* > 0.05).

**Table 3.** Group-wise average overall coupling for the whole connectome and the default mode network across groups and structural connectivity metrics

Whole connectome								
	FD–FC coupling		FbC–FC coupling		FDC–FC coupling		Streamline–FC coupling	
	*r*	*p*	*r*	*p*	*r*	*p*	*r*	*p*
AD	0.24	<0.001*	0.26	<0.001*	0.24	<0.001*	0.40	<0.001*
MCI	0.24	<0.001*	0.20	<0.001*	0.24	<0.001*	0.41	<0.001*
CN	0.24	<0.001*	0.19	<0.001*	0.24	<0.001*	0.39	<0.001*
Default mode network								
AD	0.27	0.012*	0.07	0.555	0.25	0.021*	0.43	<0.001*
MCI	0.34	0.002*	0.12	0.272	0.32	0.003*	0.46	<0.001*
CN	0.31	0.004*	0.06	0.613	0.29	0.008*	0.41	<0.001*

*Note*. AD = Alzheimer’s disease. MCI = mild cognitive impairment. CN = cognitively normal. FD = fiber density. FC = functional connectivity. FbC = fiber-bundle cross-section. FDC = combined fiber density and fiber-bundle cross-section. *p values are statistically significant (*p* < 0.05) after correction for false discovery rate.

### Edge-Wise Coupling

**Figure 3.**
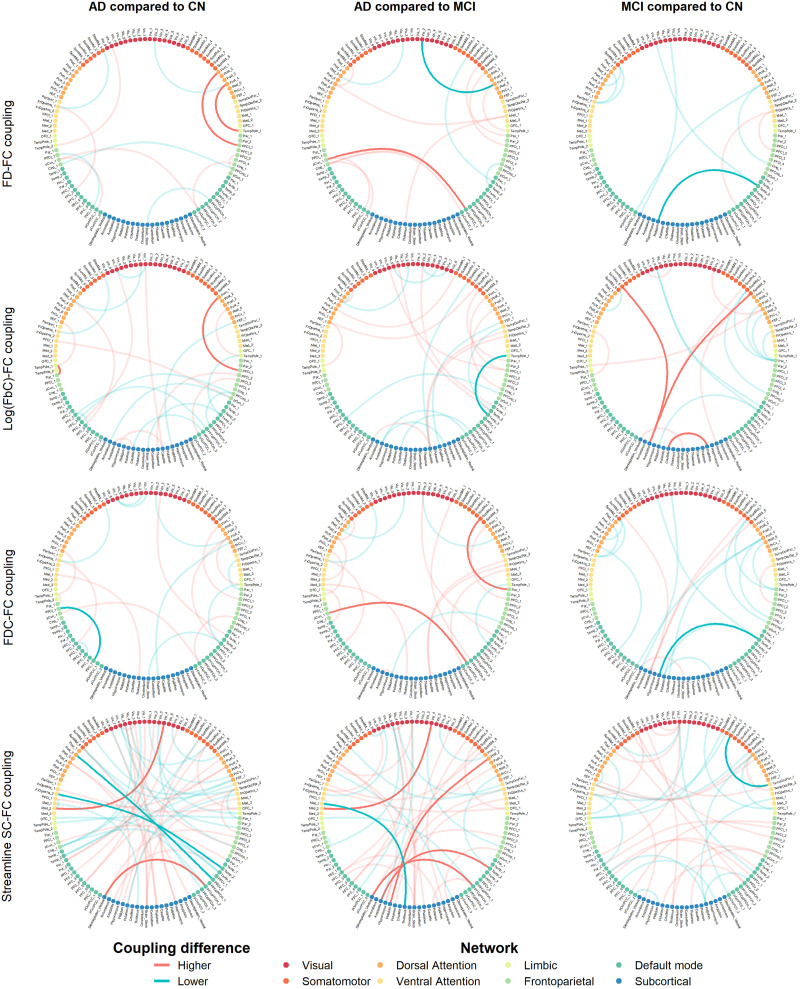
Edge-wise differences in coupling across groups and structural connectivity metrics. Graphs generated using FCtools R toolbox (https://github.com/CogBrainHealthLab/FCtools/). Higher = Large *r* value relative to the group AD or MCI is compared to. Lower = Smaller *r* value relative to the group AD or MCI is compared to. Each connecting line represents an edge, where a significant difference between groups in SC–FC coupling was observed. Coupling was defined as the Pearson *r* correlation between the functional weight and structural weight of an edge calculated across the whole group (one SC and one FC weight per participant). Using 5,000 permutations, low opacity edges were significant at *p* < 0.0025 (*p* < 0.01 with Bonferroni correction for four coupling measures), and high opacity edges that survived FDR correction across all edges of all comparisons (corrected *p* < 0.05).

The significant edges across SC metrics and groups are depicted in Figure 3. There was limited overlap in edges that demonstrated significantly different SC-FC coupling at *p* < 0.0025 between groups across different fixel-metrics: in AD compared with CN, of all significant edges, 9% overlapped across log(FbC)–FD, 31% across FD–FDC, 28% across log(FbC)–FDC; in MCI compared with CN, 12% across log(FbC)–FD, 41% across FD–FDC, 33% across log(FbC)–FDC. A limited overlap between streamline SC and fixel-wise SC, only with FDC, and for 2.9% of edges differing in AD. There was no overlap between clinical groups; none of the significant edges were commonly altered in both groups before FDR correction.

Compared with CN, robust coupling decreases involved regions of the DMN in both clinical groups, to areas in the left superior frontal sulcus in AD, the right lateral/polar temporal lobe in both AD and MCI (Figure 3); although connecting from the DMN to different regions: decoupled edges connected to the frontoparietal network (left middle dorsolateral PFC, FDC) and dorsal and ventral attention networks (left supramarginal gyrus and left insula/frontal operculum (superior part), streamline SC), in AD. For MCI, decoupled edges connected DMN regions to the left pallidum (FD, log(FbC)). An additional decrease existed in streamline SC, for an edge between the right parietal operculum/inferior parietal lobule and the inferior part of the right somatomotor cortex in MCI. Edge-wise coupling increases relative to CN affected non-DMN network for fixel metrics and involved ROIs from Yeo-7’s frontoparietal (left inferior parietal-occipital area, right inferior lateral prefrontal cortex), limbic (left temporopolar/rhinal area), dorsal attention (right lateral temporooccipital cortices) in log(FbC) and FD for AD. For MCI, increases were found connecting subcortical regions (left amygdala, left caudate nucleus, right pallidum) and bilateral somatomotor cortices in log(FbC) for MCI. Additional streamline SC coupling increases connected the right dorsomedial PFC and the left hypothalamus, the left paracentral lobule and right inferior occipital lobe in AD.

### Node-Wise Coupling

**Figure 4.**
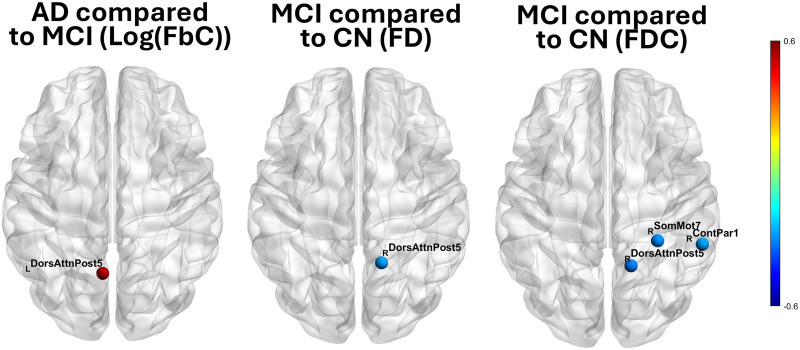
Significant node strength coupling differences across groups and structural connectivity metrics. The graphs were produced with the BrainNet Viewer MATLAB toolbox (Xia et al., 2013). Values are raw difference in coupling *r*. Each colored dot represents a network node, where a significant difference between groups in SC–FC coupling was observed. Coupling was defined as the Pearson *r* correlation between the functional nodal strength and structural nodal strength of a node across the whole group (one SC and one FC nodal strength value per participant). Using 5,000 permutations, all nodes were significant at *p* < 0.0025 (*p* < 0.01 with Bonferroni correction for four coupling measures). None survived FDR correction at *p* < 0.05.

All nodes where structural and functional node strength coupling significantly differed across groups at *p* < 0.0025, for each SC measure, are depicted in Figure 4. AD did not significantly differ in node coupling compared with CN, and had increased coupling in the left superior precuneus compared with MCI (log(FbC)). In MCI compared with CN, all nodal coupling differences occurred because of decreased coupling in MCI, affecting the right superior precuneus (FD, FDC), the superior part of right somatomotor cortex/postcentral sulcus, and the right inferior parietal lobule (FDC). No node-level differences were observed in streamline SC–FC coupling. No node-wise difference survived FDR correction.

### DMN and Cognitive Function

**Figure 5.**
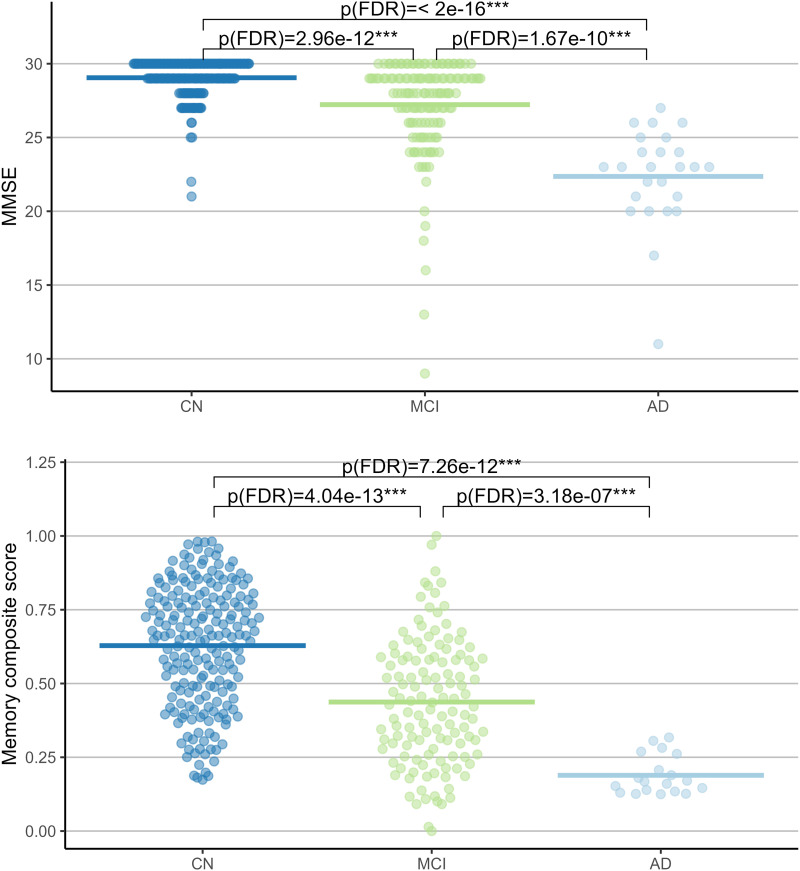
Neuropsychological outcome across groups. ***Comparisons significant at *p* < 0.001 after correction for false discovery rate, tested using Wilcoxon rank-sum tests.

The memory composite score showed a good fit for the latent variable estimation (*X*^2^ = 3.220, CFI = 0.997, root mean square error of approximation (RMSEA) = 0.042; 90% confidence interval [<0.001, 0.123]; standardized root mean square residual (SRMR) = 0.012). MMSE score significantly differed across groups, ANOVA: *F*(2) = 103.7, *p* ≤ 0.001), all groups differing from each other in Tukey’s honestly significant difference (HSD) post hoc test. The memory composite score also significantly differed across groups, *F*(2) = 64.55, *p* = < 0.001), all groups also differing from each other in Tukey’s HSD post hoc test. Both outcomes are depicted in Figure 5.

**Figure 6.**
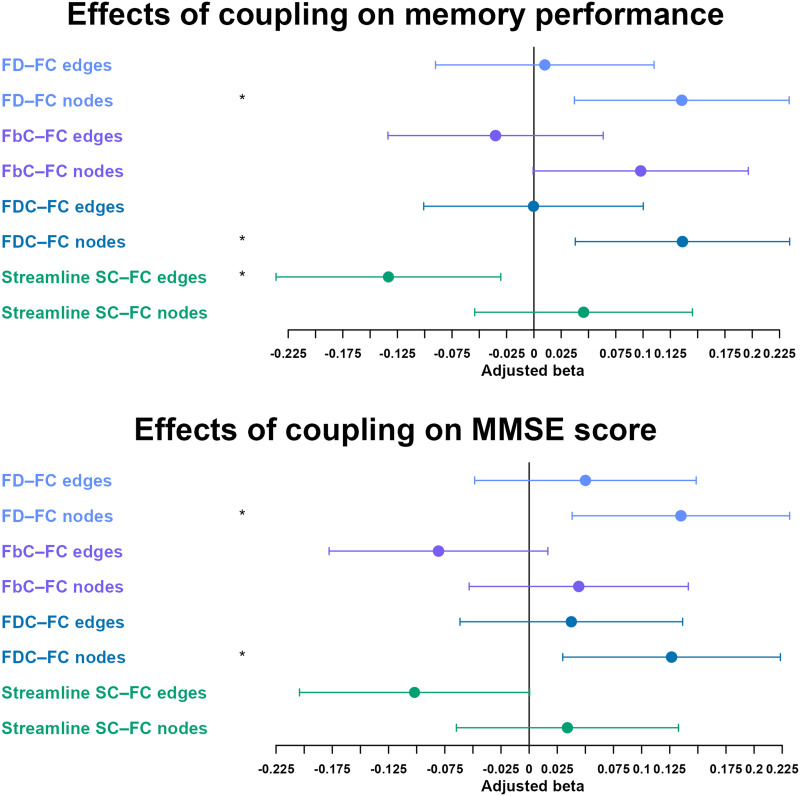
The effect of default mode network edge-wise and node-wise coupling on memory performance and Mini-Mental State Examination scores across fixel-based metrics. *Significant at *p* = < 0.05 after correction for false discovery rate. Each bar and dot represent respectively the confidence interval and standardized beta corresponding to the effect of the predictor named on the left. Edge-wise coupling and node-wise coupling were grouped together according to their SC measure (FD, FbC, FDC, streamline) in separate univariate linear models; with age, sex, and intracranial volume as control variables. Coupling of “edges” was defined with Pearson *r* correlations between the FC and SC weights of all within-DMN edges in each participant, and coupling of “nodes” was defined with Pearson *r* correlations between the FC and SC nodal strengths of all DMN nodes in each participant.

Overall coupling of DMN nodal strength significantly predicted memory performance and the MMSE score for FD–FC and FDC–FC coupling (Figure 6), with a negative effect for edge-wise streamline SC–FC coupling. Exploratory comparisons for subgroups separately show that memory composite effects were still significant in CN alone for FD and FDC, but not MMSE, with a negative log(FbC)–FC edge-wise predictive effect instead (Supporting Information S2). For coupling only including positive FC edges, edge-wise Streamline SC negatively predicted memory performance, while for coupling only including negative FC edges, no effects were found (Supporting Informations S3 and S4).

## DISCUSSION

The present study examined structure–function coupling in AD, MCI, and CN older adult participants using various approaches to estimating SC. Unlike previous studies that examined DWI-based SC–FC coupling, we studied fixel-based SC–FC coupling metrics, at the edge and node levels. The partial overlap in significant group differences across the different types of fixel-based SC–FC coupling (i.e., using FD, FDC, log(FbC)) suggests that fixel-based microscopic and macroscopic features of white matter provide distinct information about structure–function coupling. Nevertheless, overall coupling remained low to medium, not exceeding the correlation values of previously reported SC–FC coupling based on conventional connectomes (R. Cao et al., 2020; Sun et al., 2014; J. Wang et al., 2018). Additionally, we found evidence that FD–FC and FDC–FC coupling of DMN nodal strength predicted memory performance and cognitive decline for the whole group.

### Whole-Connectome Overall Coupling

The subject-wise and group-wise overall coupling results confirmed past findings of a correlation between SC and FC across the whole connectome (R. Cao et al., 2020; Dai et al., 2019; Sun et al., 2014; J. Wang et al., 2018). While Dai and colleagues did not find a significant difference in group-wise overall coupling between AD and HC (Dai et al., 2019), other studies reported a group effect, one with a significantly lower overall coupling in AD compared with both HC and MCI (Sun et al., 2014), but the opposite in two studies (R. Cao et al., 2020; J. Wang et al., 2018). No significant differences could be found for group-wise overall SC–FC coupling here, for fixel metrics nor streamline SC. These studies operationalized SC differently (e.g., streamline number for Sun et al., streamline number divided by the sum of the ROI-pair surface area in Cao et al., and a measure of density accounting for surface areas and fiber length in Dai et al. [2019]) which may partly explain the heterogeneous results. The present range of subject-wise overall coupling is comparable with ranges reported by Sun et al. (2014), and Cao et al.’s plotted individual correlations (R. Cao et al., 2020). However, group-wise overall SC–FC coupling remained low for fixel metrics, while it reached 0.41 for streamline SC, closer to the reported 0.48 in Sun et al. (2014) and J. Wang et al. (2018). Streamline-based connectivity also relied on the number of streamlines for the former (Sun et al., 2014), while a weight based on the probability of streamline assignment for the latter (J. Wang et al., 2018). The latter measure may covary more strongly with FC than fixel-based SC at a group level for whole-connectome coupling.

### Coupling Alterations in the DMN

For DMN overall coupling, significant differences were found between groups although not surviving FDR correction, with AD being decreased compared with MCI and CN, which conflicts with Dai and colleagues’ identification of an increased overall coupling in AD compared with controls in a “module” of connections within the DMN (Dai et al., 2019). At the edge level, coupling decreased compared with CN in connections to DMN regions in MCI and AD as well. Robust increases relative to CN were only found in edges not affecting the DMN for fixel metrics, which may suggest connections in other networks may manifest as a compensation to disrupted DMN coupling. In AD, decreased within-DMN edges that did not survive correction for multiple comparisons also connected bilateral DMN mPFC nodes (FDC, log(FbC)). Overall, these findings suggest fixel-based SC–FC coupling decreases in the DMN among individuals with AD. For streamline SC, one edge to the DMN was increased in AD, from the right dorsomedial PFC to the left hypothalamus, which departs from the other modalities. Such patterns are less evident when only accounting for positive or negative FC edges in isolation, with one overall within-DMN difference for positive edges (increased coupling in MCI over CN for Streamline SC), one to two increased SC–FC DMN edge in AD, and a mix of increases and decreased DMN edges in MCI (Supporting Informations S3 and S4).

### Coupling Differences Across Measures of Structural Connectivity

Distinct patterns across fixel metrics were observed in edge-wise SC–FC coupling, with minor overlap across coupling measures. The proportion of overlaps in FD and log(FbC)’s significantly altered edges, relative to their combined measure FDC, was roughly equivalent, but markedly smaller between the FD and log(FbC) measures, which implies that they reflect two distinct features of structure–function coupling and may contribute to it in different manners. Streamline SC edge-wise coupling differences generally did not overlap with any fixel-based SC measures, except with 2.9% of FDC edges, and did not detect any significant node-level coupling differences. The weight of the edges driven by streamline attribution may not reflect well structural WM “integrity” of the same edges as estimated by the FBA (Smith et al., 2022). Furthermore, non-SIFT filtered tractography, as in previous coupling studies, is subject to overdefinition of streamline seeds arising from larger WM volumes, and favors straightest paths of fiber orientation, not accounting for the total diffusion signal, which improves the accuracy of streamline assignment (Smith et al., 2013; Yeh et al., 2016). Overdefinition of streamlines likely explains the higher average number of connected edges in the streamline SC matrices, and the higher number of significantly different edges in Figure 3. Seventy-three out of the 130 significantly different edges (before FDR correction) did not exist in the SIFT-filtered tractography, and were thus potentially spurious or not consistent enough in the sample to be estimated from the representative FOD template.

### Coupling Differences Across Diagnosis Groups

Regarding patterns across diagnoses, SC–FC coupling did not differ in the same nodes and edges, which suggests coupling may not be developing in a continuous manner across the two neurological conditions. A nonlinear continuity in coupling (i.e., coupling alterations not following a continuous trend across MCI and AD) is consistent with a previous report of FC fluctuating with increases in MCI and decreases in AD and vice versa in the DMN (Yao et al., 2021). Such patterns were specifically in regions connecting to the MTL and DMPFC, two supporting subsystems of the DMN associated with memory and cognitive decline (Yao et al., 2021). At node-level, the bilateral superior precunei (DorsAttn_Post_5) also had different directions of decoupling changes between AD and MCI. We may speculate similarly to Yao and colleagues (Yao et al., 2021), that such noncontinuous alterations across MCI and AD reflect compensation mechanisms that falter as the disease progresses, requiring the recruitment of alternative neural pathways. Increased SC–FC coupling has also been suggested to reflect a lower tendency for functional network reorganization due to decreasing SC integrity (R. Cao et al., 2020; Dai et al., 2019). A compensatory interpretation remains questionable as exploratory inspection of the current data did not suggest that, for example, edges hypercoupled in MCI (not in AD), had altered SC in AD, which could explain why they are no longer oversolicited in a later disease stage. At any rate, verifying a disease progression or compensatory reorganization would require longitudinal analyses.

### DMN Coupling Measures Predicting Cognitive Outcome

DMN FD–FC and FDC–FC node-wise coupling significantly predicted memory and MMSE scores. This is in line with the results reported by Cao and colleagues, which found coupling metrics significantly predicted the MMSE and the Montreal Cognitive Assessment in MCI and AD (R. Cao et al., 2020). The fact that only FD and FDC coupling metrics were robustly associated with cognition suggests node coupling is more relevant to cognition at the microstructural level of WM, while SC metrics characterizing macrostructural features such as log(FbC) may not be as relevant. The “raw” streamline SC coupling, predicting instead a decrease in memory score at the edge level, suggests that this type of SC reflects a different construct from the fixel-based description of WM. It is a possibility that DMN nodes could contribute to effects reported by Cao and colleagues for general non-within-rich clubs edges (R. Cao et al., 2020). Given only a minority of rich clubs were part of the DMN in the latter, it is plausible that edges not connecting rich clubs that were associated with cognition are similar to the DMN nodes’ edges tested in the present study. Moreover, the fact that fixel-based coupling of all within-DMN edges did not predict any outcome measure, while the nodal coupling did, may be explained by the fact that node strength calculation was *not* restricted to within-DMN edges, which potentially means that especially internetwork connections (from DMN to other networks) support cognitive performance. That cognitive performance relied particularly connections to other networks rather that intra-DMN may also explain the negative effect of the streamline SC–FC coupling for within-DMN edges. Given previous studies have also shown that internetwork FC also relate to amyloid beta presence and cognitive decline (Elman et al., 2016; Van Hooren et al., 2018), future coupling studies may need to investigate such connections. In conclusion, the SC–FC coupling of DMN nodes in the neurocognitive aging context may also be a contributing factor to cognitive function in addition to network FC on its own (Celebi et al., 2016; Weiler et al., 2014; F. P. G. Yang et al., 2021).

### Interpreting Negative Coupling

Negatively coupled edges or nodes may be explained by several factors, including the fact that many nodes do not co-activate/deactivate highly during the resting state, as opposed to during specific tasks for which task-based networks may be recruited. FC is not majorly influenced by FD or bundle cross-section and vice versa, hence the low overall fixel-based SC–FC coupling. Moreover, averaging the fixel metrics across fixels of a given edge dilutes the SC in the same edge: sections of the same tract where FD, FbC, or FDC may differ, especially for tracts comprising many fixels, may not be meaningful for coupling. There are other explanations pertaining to the clinical populations. For instance, reduced FC in AD can be found in the absence of visible white matter alterations (Balachandar et al., 2015). When asynchronous SC or FC alterations happen (R. Cao et al., 2020; Dai et al., 2019; Sun et al., 2014), it is plausible that larger bundles are most affected by a decrease in functional activity, leading to negative coupling. Conversely, given that increased FC has been interpreted in AD as a possible “overload” of the neuron’s metabolic activity due to higher processing demands and inefficient neuronal communication (Jones et al., 2016), structural connections with fewer fibers may end up more functionally solicited in the clinical groups.

### Strengths and Limitations

This study is the first to analyze structure–function connectome coupling using fixel-based metrics in the aging population to our knowledge. This is the main strength of this study as FBA allows one to study the structural connectome with different WM features/characteristics. The study was however limited by the relatively low *b* values of the diffusion-weighted images: while single-shell data from the ADNI3-Basic protocol allowed us to maximize sample size, this came at the expense of lower data quality compared with high *b* values multishell data, thus limiting the accuracy of the fixel estimations (Raffelt et al., 2012). As such, spuriously high FD or log(FbC) could bias the averaging of the fixels along masks that were used to define SC edges. This averaging approach is a limitation as the strength of the SC–FC coupling may also differ across fixels along a tract. A proposed measure of “fibre bundle capacity” may also be more effective in capturing accurate connectivity, while implementing information from FD and FbC as part of the tractography (Smith et al., 2022) available with the “SIFT2” algorithm, not yet compatible with MRTRIX3’s FBA pipeline. Fundamentally, however, structure–function correlation does not prove the possible underlying mechanism, and studies at a cellular or microscropic scale may be able to explain further the relationship between FC and WM features, especially when it comes to speculated dynamic reorganization (R. Cao et al., 2020; Dai et al., 2019).

More broadly, coupling as a correlation between matching direct SC and FC connections, although a conventional approach (R. Cao et al., 2020; Sun et al., 2014), may neglect “relay” connections that together, could contribute to the FC between a pair of regions. Another aspect is that previous structure–function coupling analyses reported that coupling may vary across time windows of an fMRI recording, influencing the topology of the FC network (Fukushima et al., 2018), while the static FC presently used only represents an average network topology in a session. Furthermore, the analyses were limited by the cross-sectional nature of the data: calculating coupling across group (computing one correlation per edge or node) was the most viable approach for edge or node-specific analyses, as only one pair of SC and FC observation could be matched for a given subject. It may be more meaningful to look at individual coupling over time to obtain individual edge-specific and node-specific coupling values. This is especially true for edge-specific effects on cognition (Weiler et al., 2014; Zhou et al., 2022), which could not be tested with DMN coupling due to the single-observation nature of the data.

### Conclusion

This study used FBA to investigate distinct WM features in association with FC in the neurocognitive aging spectrum. While structure–function coupling across the whole connectome was not different across groups, overall coupling within the DMN network was decreased in AD. Edges and nodes had significantly altered coupling in AD and MCI relative to CN, with no significantly altered edge or node common to both groups. Furthermore, these AD- and MCI-related alterations did not occur in a manner that is consistent with the severity of cognitive impairment. Edge-wise coupling decreased significantly within DMN regions in MCI and AD, compared with CN, whereas edge-wise coupling in MCI and AD was significantly increased beyond the DMN, reinforcing the idea that coupling alterations reflect a dynamic reorganization of brain networks. Finally, SC and FC coupling of DMN node strengths predicted MMSE and verbal memory scores in the whole group, specifically for FDC and FD measures, which reflect a more prevalent contribution of microstructural WM features.

## SUPPORTING INFORMATION

Supporting information for this article is available at https://doi.org/10.1162/netn_a_00461. Code for the coupling analyses is available at https://github.com/chabld/FBA_coupling. Data collection and sharing for this project was funded by the Alzheimer's Disease Neuroimaging Initiative (ADNI) (National Institutes of Health Grant U01 AG024904) and DOD ADNI (Department of Defense award number W81XWH-12-2-0012). ADNI is funded by the National Institute on Aging, the National Institute of Biomedical Imaging and Bioengineering, and through generous contributions from the following: AbbVie, Alzheimer’s Association; Alzheimer’s Drug Discovery Foundation; Araclon Biotech; BioClinica, Inc.; Biogen; Bristol-Myers Squibb Company; CereSpir, Inc.; Cogstate; Eisai Inc.; Elan Pharmaceuticals, Inc.; Eli Lilly and Company; EuroImmun; F. Hoffmann-La Roche Ltd and its affiliated company Genentech, Inc.; Fujirebio; GE Healthcare; IXICO Ltd.; Janssen Alzheimer Immunotherapy Research & Development, LLC.; Johnson & Johnson Pharmaceutical Research & Development LLC.; Lumosity; Lundbeck; Merck & Co., Inc.; Meso Scale Diagnostics, LLC.; NeuroRx Research; Neurotrack Technologies; Novartis Pharmaceuticals Corporation; Pfizer Inc.; Piramal Imaging; Servier; Takeda Pharmaceutical Company; and Transition Therapeutics. The Canadian Institutes of Health Research is providing funds to support ADNI clinical sites in Canada. Private sector contributions are facilitated by the Foundation for the National Institutes of Health (www.fnih.org). The grantee organization is the Northern California Institute for Research and Education, and the study is coordinated by the Alzheimer’s Therapeutic Research Institute at the University of Southern California. ADNI data are disseminated by the Laboratory for Neuro Imaging at the University of Southern California.

## AUTHOR CONTRIBUTIONS

Charly Hugo Alexandre Billaud: Data curation; Formal analysis; Investigation; Methodology; Software; Validation; Visualization; Writing – original draft; Writing – review & editing. Junhong Yu: Data curation; Funding acquisition; Methodology; Project administration; Resources; Software; Validation; Writing – review & editing.

## FUNDING INFORMATION

Junhong Yu, Nanyang Technological University (https://dx.doi.org/10.13039/501100001475), Award ID: 021080-00001.

## Note

^1^ Note: This is a corrected article. See the details of the correction here: https://doi.org/10.1162/NETN.x.506.

## Supplementary Material



## TECHNICAL TERMS

Default mode network: A set of connected brain regions that are active when an individual is at rest and not doing a specific action.
Mild cognitive impairment: A neurocognitive disorder involving cognitive decline distinct from normal aging, but not severe enough to be classified as dementia.
Structure–function coupling: The statistical association (often correlation) between structural connectivity and functional connectivity.
Diffusion-weighted imaging: A method of brain imaging that measures the motion of water molecules within tissues.
Fixel: A neural fiber population inside a voxel.
Fiber density: Measure of white matter microstructure, approximate to the intra-axonal volume fraction.
Fiber-bundle cross-section: Measure of white matter macrostructure and morphology, local volumetric differences relative to a template space.
Fiber density and cross-section: The combined product of fiber density and fiber-bundle cross-section.
Streamline: A three-dimensional line representing the path of a fiber population.
Connectome: a network of connections (in this context, connections between pairs of brain regions).
Declarative memory: the conscious acquisition and recollection of information about facts, data, or events.
Fiber orientation distribution: information about the orientation of fiber populations and their volume fractions.
