# Erratum: Structure–function coupling using fixel-based analysis and functional magnetic resonance imaging in Alzheimer’s disease and mild cognitive impairment

**DOI:** 10.1162/NETN.x.506

**Published:** 2025-12-01

**Authors:** Charly Hugo Alexandre Billaud, Junhong Yu

**Affiliations:** Nanyang Technological University, Psychology, School of Social Sciences, 48 Nanyang Avenue, Singapore 639798, Singapore

The article was updated due to a mistake that affected a portion of the results in the original article. The mistake concerned a script used for statistical analyses and results reported for the “Streamline Structural Connectivity (SC)-Functional Connectivity (FC) coupling” specifically. The streamline SC–FC coupling modality was not fundamental or a subject of our hypotheses but added a conventional method to be put in parallel with our central, novel methods (fixel-based coupling). While we did not directly compare them in the article, they needed to be accurately reported. The fixel-based coupling modality results, the main focus and foundation of the article, remained unaffected. Similarly, the code shared publicly for the computation of the coupling was left untouched as it pertained to a later stage of the analysis.

**Description of the corrected error:**

The script converted each individual matrix into a vector of 7,021 vertices (representing the 119 × 119 connections), but an option was forgotten that led to the first row of each matrix to be interpreted as column names instead of the first row of numbers (matrices became 118 × 119). Unfortunately, vectors of the right dimension were still generated with no error message (silently producing “NA” cells where adjacency connections went missing), which left the error unnoticed until later scrutiny. This only affected the streamline-count matrices as they were computed in a separate script from the fixel-based matrices.

**Summary of the changes:**

Whole-brain coupling for streamline SC–FC coupling, while already noted to be high, is now higher than previously reported, at correlations that are more in line with cited literature. Changes also further confirm, as discussed, that Alzheimer’s disease (AD) and mild cognitive impairment (MCI) do not follow a continuous or “linear” pattern of coupling differences compared with cognitively normal (CN) controls, with an increased overall coupling in MCI, not observed in AD. There is now a very small overlap between differing edges in fixel-based modalities and the streamline count instead of none. Streamline SC coupling is now shown to predict memory performance negatively, as opposed to the fixel-based findings. Edges that differed across groups in that modality are now located differently and one edge no longer applies as a discussed example of MCI–AD discontinuity, while not affecting overall conclusions about the latter theme.

**Specific details of corrections and locations in the article:**

- Higher whole-brain coupling than previously shown for streamline SC–FC coupling (Figure 2, Table 3), for example, the *r* went from 0.2 to 0.4 for the whole-brain group-average in AD (Table 2).
- Due to updates in *p* values, the significant results in Figure 2 no longer survive correction for false discovery rate (FDR) (*p* = 0.0507 after correction).
- Overall default mode network (DMN) streamline SC–FC coupling differences no longer significantly differ between AD and CN but now do for CN and MCI (Figure 2).
- Section “Coupling differences across diagnosis groups”: An edge that was increased in AD compared with CN and decreased in MCI compared with CN was discussed, but it no longer differs.
- A minor overlap of significantly different edges between one fixel metric and the streamline SC (2.9%), previously 0%.
- Section “Coupling alterations in the default mode network”: One edge to the DMN is now increased in AD (Figure 3). Our discussion about the opposite observation now only applies for the fixel-based measures specifically.
- There is now a negative effect of within-DMN edges on memory performance for streamline SC–FC coupling (Figure 6, discussed in “Default mode network coupling measures predicting cognitive outcome”).

Supporting information:

- S1: Streamline SC–FC coupling now differs across groups for Yeo-7’s ventral attention network, not the dorsal attention or limbic networks (Table S1). None survive FDR correction (Figure S1).
- S1: Subnetworks’ overall subject-wise coupling are also relatively higher than previously reported for streamline SC–FC coupling (Table S2).
- S2: Figure S2 was updated as it contained the main text’s result (cognitive analyses for “All” participants) but otherwise did not change.
- S3: There is now a higher than previously reported overall coupling for streamline SC–FC coupling (Figure S3, Table S3).
- S3: There is a significant increase in overall DMN streamline SC–FC coupling in MCI compared with CN (Figure S3).
- S3: There is now a significant effect of diagnosis on subject-wise whole-connectome streamline SC–FC coupling (pairwise post hoc comparisons: AD < MCI, not significant after FDR correction) and on DMN SC–FC coupling with a significant pairwise comparison (CN < MCI, surviving correction).
- S3: Figure S4 and S5 now report differing edges and nodes at other locations for streamline SC–FC coupling.
- S3: Streamline SC–FC edge-wise within-DMN coupling now negatively predicts memory performance (Figure S6).
- S4: Overall coupling values were updated but no effect has become significant or lost significance (Figure S7, Table S4).
- S4: Figure S8 and S9 now report significantly differing edges and nodes at other locations for streamline SC–FC coupling.
- S4: Streamline SC–FC coupling’s effects on cognitive measures are no longer significant (Figure S10).

For reference, the corrected version of this article is available here: https://doi.org/10.1162/netn_a_00461.

