# Supplementary material for: Structure–function coupling using fixel-based analysis and functional magnetic resonance imaging in Alzheimer’s disease and mild cognitive impairment
Source: Netw Neurosci. 2025 Jul 29;9(3):969–89. doi: 10.1162/netn_a_00461 (PMC12543303; doi:10.1162/netn_a_00461)
Supplement: Supplementary file 1 [file netn-9-3-969-s001.pdf]

S1. Group differences in overall coupling for all networks

The subject-wise overall coupling, for edges of each of the Yeo 7 networks and subcortices from the aseg segmentations, across groups and SC metrics, are depicted in Figure S1. For each network, the effect of diagnosis group on overall coupling is reported in Table S1.

Table S1. Effect of diagnosis group on subject-wise overall coupling, controlling for age and sex, for each network separately

	**Effect of diagnosis group on coupling per SC measure**								
	**FD**		**FbC**		**FDC**		**Streamline**		
**Network**	***F***	***p***	***F***	***p***	***F***	***p***	***F***	***p***	**Post-hoc test**
**Visual**	2.358	.096	1.219	.297	1.936	.146	0.162	.851	NA
**Somatomotor**	3.225	.041*	1.813	.165	3.544	.030*	1.812	0.165	FD, FDC: MCI>CN*
**Dorsal Attention**	1.102	.302	2.802	.062	1.064	.346	1.259	0.285	NA
**Ventral Attention**	1.419	.243	2.494	.084	1.391	.250	3.424	.034*	Streamline: CN < MCI*
**Limbic**	1.372	.255	2.045	.131	1.430	.241	1.444	.237	NA
**Frontoparietal**	0.976	.378	0.068	.935	0.788	.456	2.613	.075	NA
**Subcortical (aseg)**	0.767	.465	4.474	.012*	0.926	.397	2.512	.082	FbC: n.s.

Note. AD=Alzheimer's disease. MCI=Mild cognitive impairment. CN=Cognitively normal. FD=Fibre density. FC=Functional connectivity. FbC=Fibre-bundle cross-section. FDC=Combined fibre density and fibre-

bundle cross-section. Post-hoc tests were pairwise contrast of marginal means. * Significant at $p < .05$ ** Significant at $p < .01$, uncorrected for multiple comparisons. Post-hoc tests were pairwise contrasts of estimates of marginal means, when a significant effect of diagnosis group existed.

Details of the post-hoc comparisons (plotted in Figure S1 when surviving per-network FDR correction) in Table S1 are as follows

- Somatomotor network: MCI had an increased coupling compared to CN (FD: t=-2.2332, p=0.02; FDC: t=-2.441, p=0.02) which did not survive FDR correction across measures of that network.
- Ventral Attention network: CN had a lower coupling compared to MCI (Streamline: t=-2.120, p=.035) which did not survive FDR correction across measures of that network.

The coupling for SC and FC group averages in each network and SC metrics are depicted in Table S2. No significant difference in group-wise coupling was detected for any SC measure for Fisher Z transform tests (all p>.05).

Table S2. Group-wise average overall coupling for each Yeo network and structural connectivity metrics

	**FD – FC coupling**		**FbC – FC coupling**		**FDC – FC coupling**		**Streamline – FC coupling**	
	r	***p***	***r***	***p***	***r***	***p***	***r***	***p***
				**Visual Network**				
**AD**	0.292	.0018*	0.26	.0056	0.289	.0020*	0.335	>.0001*
**MCI**	0.301	.0013*	0.213	.0244*	0.296	.0015*	0.326	.0001*

**CN**	0.318	.0006*	0.198	.0362*	0.311	.0008*	0.324	.0001*
				**Somatomotor network**				
**AD**	0.461	.0019*	0.391	.0096*	0.457	.0021*	0.453	>.0001*
**MCI**	0.472	.0014*	0.215	.1655	0.465	.0017*	0.437	>.0001
**CN**	0.453	.0023*	0.246	.1113	0.446	.0027*	0.422	>.0001
				**Dorsal Attention network**				
**AD**	0.303	.0480*	0.296	.0537	0.302	.0490*	0.318	.0009*
**MCI**	0.353	.0203*	0.301	.0497*	0.350	.0214*	0.311	.0013*
**CN**	0.356	.0191*	0.317	.0385*	0.352	.0205*	0.293	.0025
				**Ventral Attention network**				
**AD**	0.275	.2047	0.309	.1509	0.282	.1920	0.393	.0011*
**MCI**	0.224	.3039	0.213	.3282	0.226	.3008	0.359	.0031*
**CN**	0.21	.3366	0.227	.2978	0.211	.3336	0.320	.0089*
				**Limbic network**				
**AD**	-0.817	.0916	-0.702	.1862	-0.796	.1068	-0.164	.0650
**MCI**	-0.754	.1412	-0.62	.2647	-0.731	.1606	-0.029	.9373
**CN**	-0.761	.1354	-0.636	.2489	-0.740	.1530	-0.088	.8093
				**Frontoparietal network**				
**AD**	0.328	.1015	0.081	.6947	0.315	.1175	0.372	.0008*
**MCI**	0.36	.0709	-0.01	.9612	0.345	.0847	0.379	.0006*
**CN**	0.34	.0897	-0.05	.8093	0.325	.1057	0.324	.0039*
				**Subcortical (aseg) network**				
**AD**	0.084	.3429	0.097	.2683	0.083	.3468	0.096	.2704

**MCI**	0.117	.1817	0.017	.8450	0.117	.1833	0.094	.2763
**CN**	0.098	.2636	-0.053	.5446	0.096	.2730	0.139	.1066

Note. AD=Alzheimer's disease. MCI=Mild cognitive impairment. CN=Cognitively normal. FD=Fibre density. FC=Functional connectivity. FbC=Fibre-bundle cross-section. FDC=Combined fibre density and fibre-bundle cross-section. * Significant at $p < .05$, uncorrected for multiple comparisons.

Figure S1. Subject-wise overall coupling for the 7 Yeo networks and aseg subcortical regions across groups and structural connectivity metrics

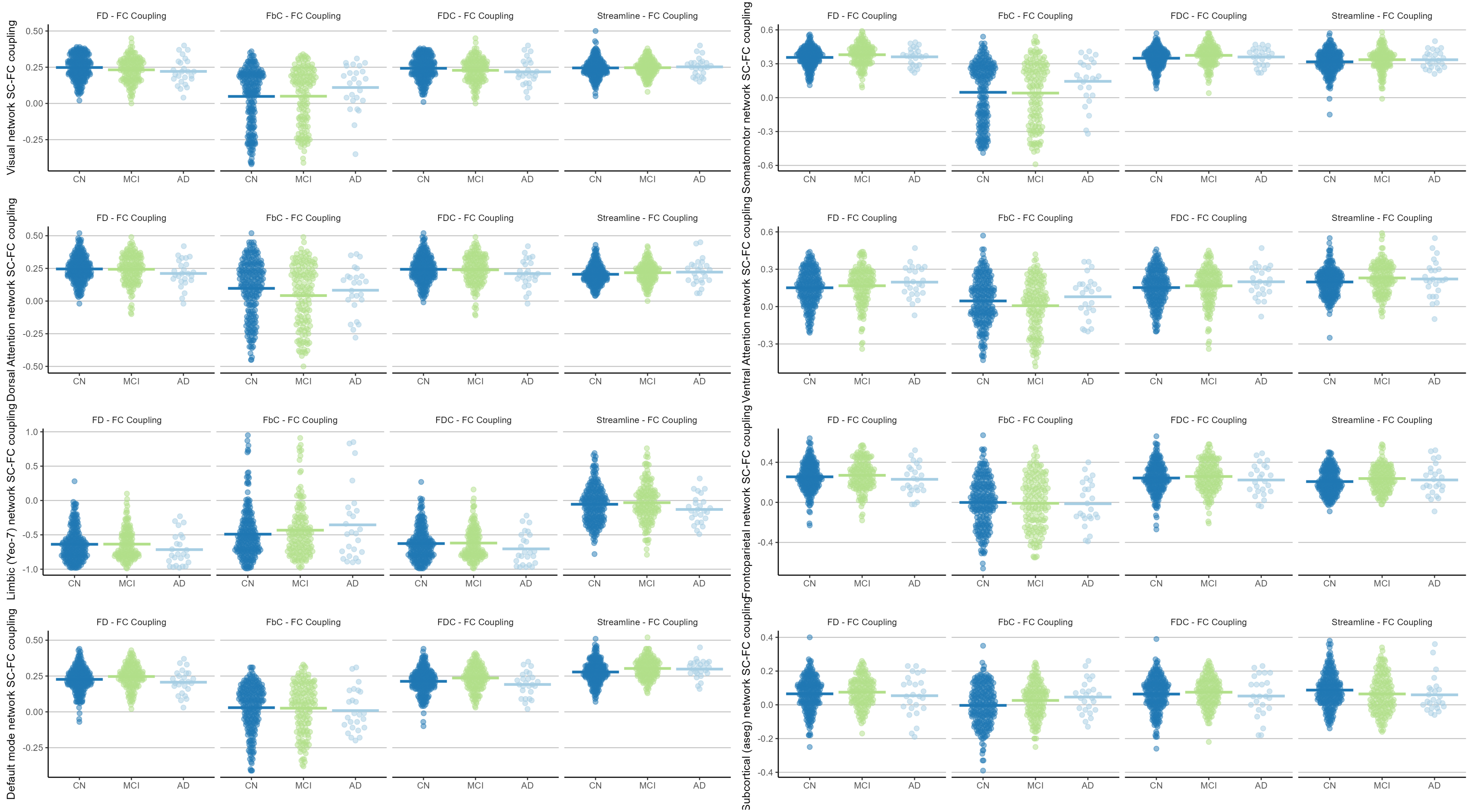

Note. DMN = Default mode network. AD=Alzheimer's disease. MCI=Mild cognitive impairment. CN=Cognitively normal. FD=Fibre density. FC=Functional connectivity. FbC=Fibre-bundle cross-section. FDC=Combined fibre density and fibre-bundle cross-section. Each dot represents a participant's Pearson r correlation

between the weights of FC and SC edges for the whole connectome (top panel) and for within-DMN edges (bottom panel). The coloured thick bars represent the group mean r. * Comparisons significant at $p < .05$ after correction for false discovery rate (across comparisons for each specific network separately, not across all networks), obtained with post-hoc contrast of estimates of marginal means.

S2. Effect of default mode network coupling on cognition in subgroups separately

Figure S2. The effect of default mode network edge-wise and node-wise coupling on memory performance and Mini-Mental Status Examination scores across fixel-based metrics for the whole dataset and each group separately

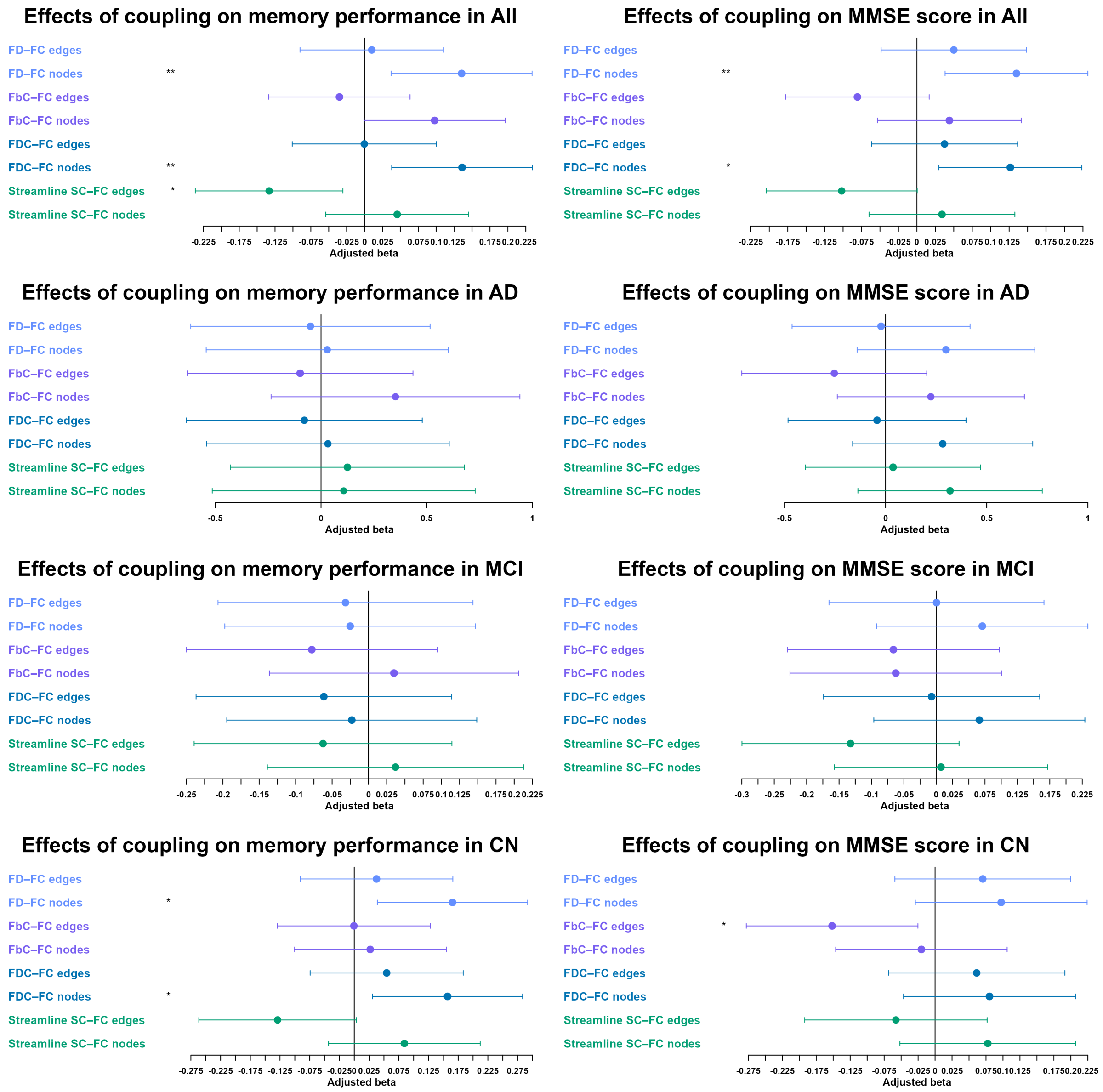

Note. AD=Alzheimer's disease. MCI=Mild Cognitive impairment. CN=Cognitively normal. FD=Fibre density. FC=Functional connectivity. FbC=Fibre-bundle cross-section. FDC=Combined fibre density and fibre-bundle cross-section. * Significant at *p*=<.05 ** Significant at *p*=<.01, uncorrected. Only the analysis for All

participants stays significant after FDR correction across predictors of each model separately. Each bar and dot represent respectively the confidence interval and standardized beta corresponding to the effect of the predictor named on the left. Edge-wise coupling and node-wise coupling were grouped together according to their SC measure (FD,FbC,FDC,streamline) in separate univariate linear models; with age, sex and intracranial volume as control variables. Coupling of "edges" was defined with Pearson *r* correlations between the FC and SC weights of all within-DMN edges in each participant, and coupling of "nodes" was defined with Pearson *r* correlations between the FC and SC nodal strengths of all DMN nodes in each participant.

S3. Coupling analysis with only positive functional connectivity edges

The following results reproduce the same coupling analysis protocol with the addition of removing *negative* functional connectivity edges from the FC matrices, thus only accounting for positively connected functional edges.

Overall whole-connectome and default mode network coupling

The subject-wise overall coupling, for edges of the whole connectome and of the DMN, across diagnosis groups and SC metrics, are depicted in Figure S3. Diagnosis group had no significant effect on subject-wise whole-connectome coupling for any SC measure (FD: F=2.085, p=.126; Log(FbC): F= 1.113, p=.330; FDC: F=1.000, p=.369) but did for streamline SC: F= 3.928, p=.020; post-hoc pairwise contrast: AD > CN at p=.629, AD < MCI at p=.619, CN < MCI at p=.052). Diagnosis group had no effect on subject-wise overall DMN coupling for FD (F= 2.687 p=.069); Log(FbC) – FC coupling (F=0.023, p=.977); It did for FDC (FDC: F=3.031, p=.0494), but post-hoc tests did not return any significant pair-wise comparison (AD < CN at p=.21; AD < MCI at p=.035; CN < MCI at p=.081; not surviving FDR correction). It also had an effect for Streamline SC (F=5.999, p=.003), with a significant post-hoc difference (CN < MCI, at p=.008).

Figure S3. Subject-wise overall coupling for the whole connectome and the default mode network across groups and structural connectivity metrics (only positive functional edges)

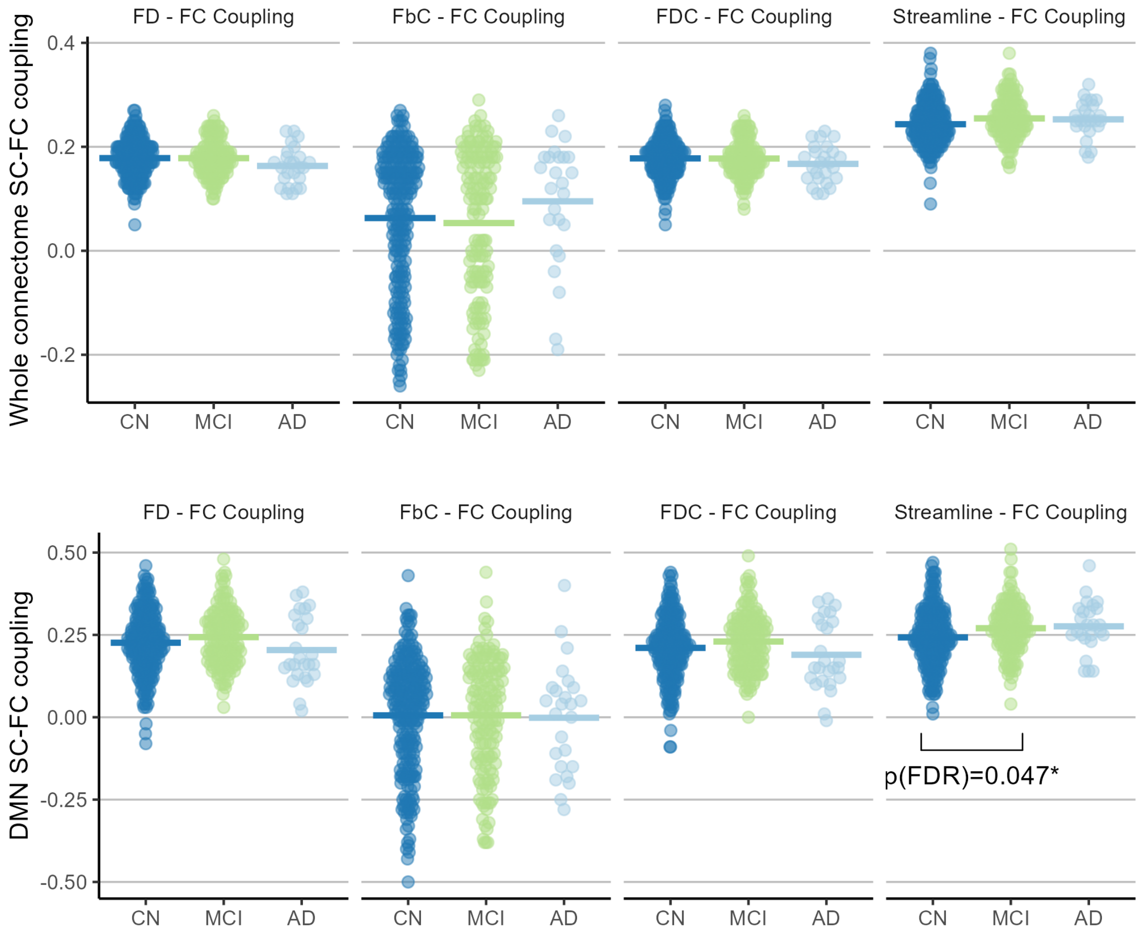

Note. DMN = Default mode network. AD=Alzheimer's disease. MCI=Mild cognitive impairment. CN=Cognitively normal. FD=Fibre density. FC=Functional connectivity. FbC=Fibre-bundle cross-section. FDC=Combined fibre density and fibre-bundle cross-section. Each dot represents a participant's Pearson *r* correlation between the weights of FC and SC edges for the whole connectome (top panel) and for within-DMN edges (bottom panel). The coloured thick bars represent the group mean *r*.

The whole-connectome and within-DMN overall coupling for group averages are depicted in Table S3. No significant difference (Fisher Z transform) in coupling was detected between groups for any SC measure, either for the whole connectome or for connections within the DMN (all $p > .05$).

Table S3 Group-wise average overall coupling for the whole connectome and the default mode network across groups and structural connectivity metrics (only positive functional edges)

	FD – FC coupling		FbC – FC coupling		FDC – FC coupling		Streamline – FC coupling	
**Whole connectome**								
	r	***p(FDR)***	***r***	***p(FDR)***	***r***	***p(FDR)***	***r***	***p(FDR)***
**AD**	0.222	>.001*	0.257	>.001*	0.229	>.001*	0.395	>.001*
**MCI**	0.236	>.001*	0.193	>.001*	0.24	>.001*	0.417	>.001*
**CN**	0.238	>.001*	0.189	>.001*	0.242	>.001*	0.400	>.001*
**Default mode network**								
**AD**	0.277	.012*	0.067	.568	0.255	.021*	0.425	>.001*
**MCI**	0.346	.002*	0.112	.337	0.329	.003*	0.442	>.001*
**CN**	0.316	.004*	0.048	.662	0.295	.008*	0.401	>.001*

Note. AD=Alzheimer's disease. MCI=Mild cognitive impairment. CN=Cognitively normal. FD=Fibre density. FC=Functional connectivity. FbC=Fibre-bundle cross-section. FDC=Combined fibre density and fibre-bundle cross-section. * p values are statistically significant ($p<.05$) after correction for false discovery rate.

Edge-wise coupling

The significant edges across SC metrics and groups are depicted in Figure S4.

Figure S4. Edge-wise differences in coupling across groups and structural connectivity metrics (only positive functional edges)

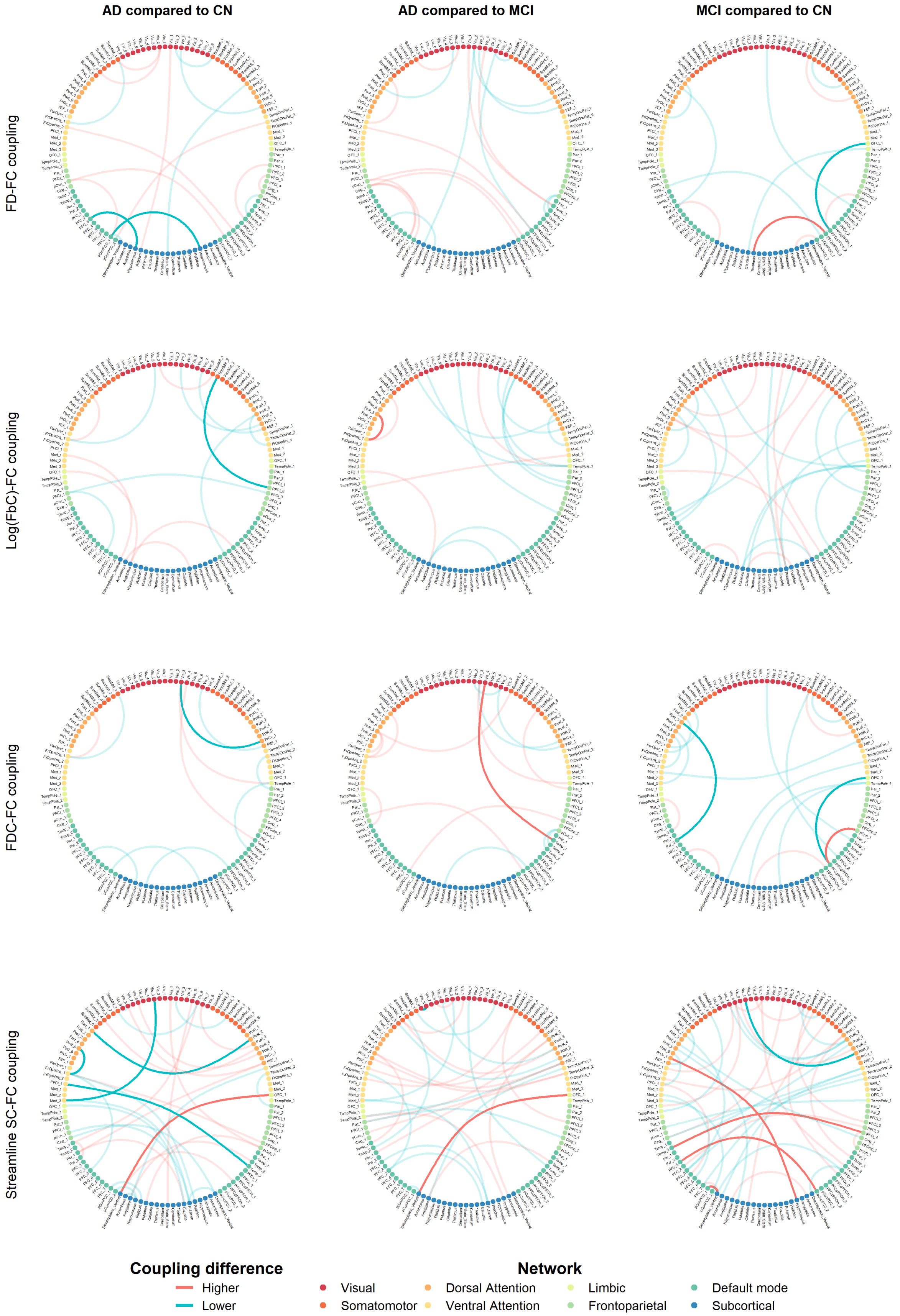

AD compared to CN
AD compared to MCI
MCI compared to CN
FD-FC coupling
Log(FbC)-FC coupling
FDC-FC coupling
Streamline SC-FC coupling
Coupling difference
Higher
Lower
Network
Visual
Somatomotor
Dorsal Attention
Ventral Attention
Limbic
Frontoparietal
Default mode
Subcortical

Note. Graphs generated using FCtools R toolbox (https://github.com/CogBrainHealthLab/FCtools/). Higher = Large *r* value relative to the group AD or MCI is compared to. Lower = Smaller *r* value relative to the group AD or MCI is compared to. AD = Alzheimer's disease. CN=Cognitively normal. MCI=Mild cognitive impairment. FD=Fibre density. FC=Functional connectivity. FbC=Fibre-bundle cross-section. FDC=Combined fibre density and fibre-bundle cross-section. SC=Structural connectivity. Each connecting line represents an edge, where a significant difference between groups in SC-FC coupling was observed. Coupling was defined as the Pearson *r* correlation between the functional weight and structural weight of an edge calculated across the whole group (one SC and one FC weight per participant). Using 5000 permutations, low opacity edges were significant at $p < .0025$ ($p < .01$ with Bonferroni correction for 4 coupling measures), and high opacity edges that survived FDR correction across all edges of all comparisons (corrected $p < .05$).

Node-wise coupling

All nodes where structural and functional node strength coupling significantly differed across groups, for each SC measure, are depicted in Figure S5. No node wise coupling difference was significant after correction for false discovery rate.

Figure S5. Significant node strength coupling differences across groups (only positive functional edges)

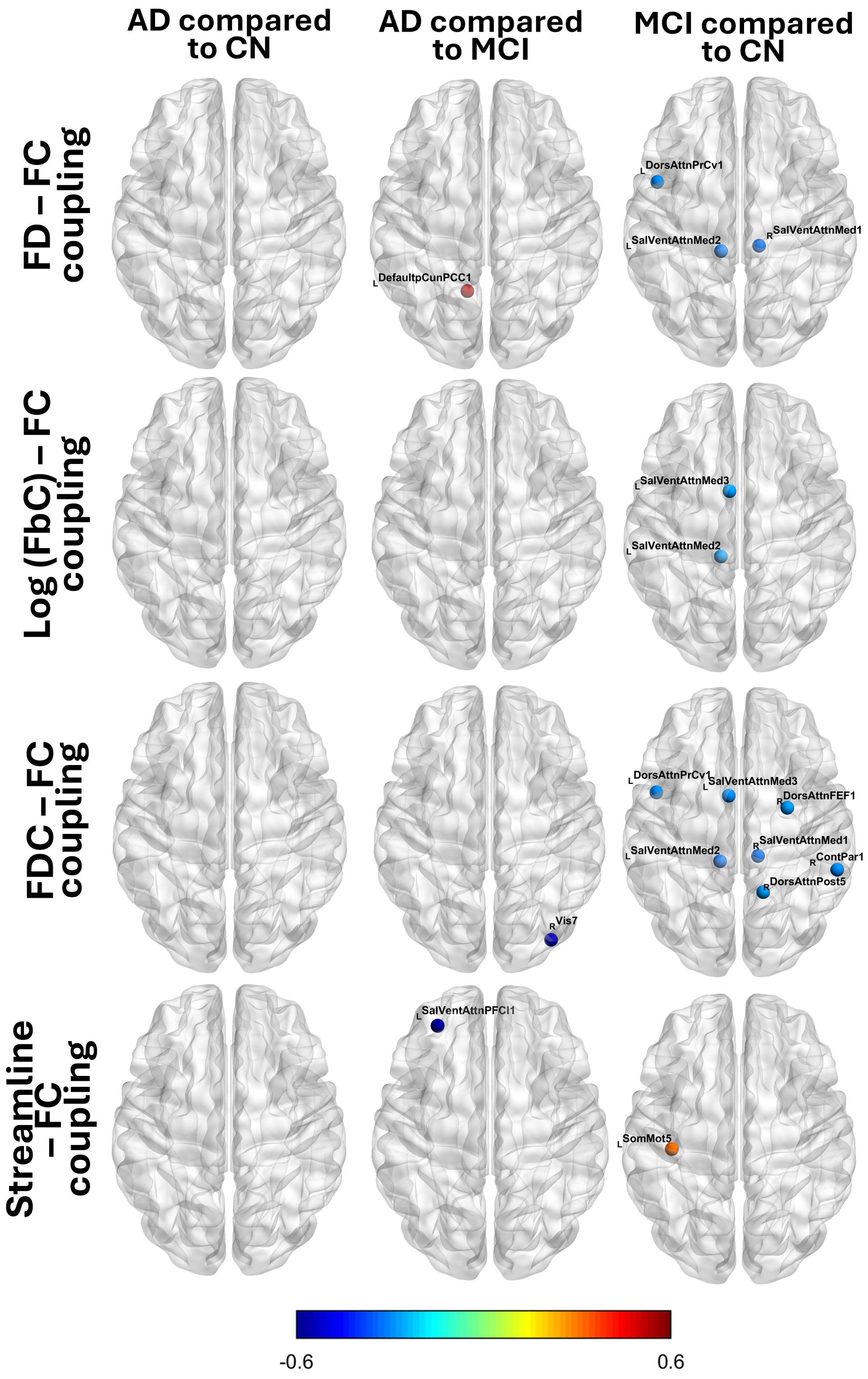
AD compared to CN
AD compared to MCI
MCI compared to CN
FD – FC coupling
Log (FbC) – FC coupling
FDC – FC coupling
Streamline – FC coupling
DefaultpCunPCC1
DorsAttnPrCv1
SalVentAttnMed2
SalVentAttnMed1
SalVentAttnMed3
SalVentAttnMed2
DorsAttnPrCv1
SalVentAttnMed3
DorsAttnFEF1
SalVentAttnMed2
SalVentAttnMed1
ContPar1
DorsAttnPost5
Vis7
SalVentAttnPFCl1
SomMot5
-0.6
0.6

Note. The graphs were produced with the BrainNet Viewer MATLAB toolbox (Xia et al., 2013). Values are raw difference in coupling *r*. AD = Alzheimer's disease. CN=Cognitively normal. MCI=Mild cognitive impairment. FD=Fibre density. FC=Functional connectivity. FbC=Fibre-bundle cross-section. FDC=Combined fibre density and fibre-bundle cross-section. Each coloured dot represents a network node, where a significant difference between groups in SC-FC coupling was observed. Coupling was defined as the Pearson *r* correlation between the functional nodal strength and structural nodal strength of a node across the whole group (one SC and one FC nodal strength value per participant). Using 5000 permutations, all nodes were significant at $p < .0025$ ($p < .01$ with Bonferroni correction for 4 coupling measures). None survived FDR correction at $p < .05$.

Default Mode Network and cognitive function

Streamline SC-FC edge-wise coupling negatively predicted memory performance (Figure S6). Overall coupling of DMN nodal strength did not significantly predict memory performance or MMSE score (Figure S6). Before FDR correction, significant predictors of MMSE at $p < .05$ were Log(FbC)-FC edge-wise coupling in CN (p=0.044, β=-0.140); and Streamline SC-FC node-wise coupling (p=.014, β=0.122) and edge-wise coupling (p=.032, β=-0.109) in all participants.

Figure S6. The effect of default mode network edge-wise and node-wise coupling on memory performance and Mini-Mental Status Examination scores across fixel-based metrics (only positive functional edges)

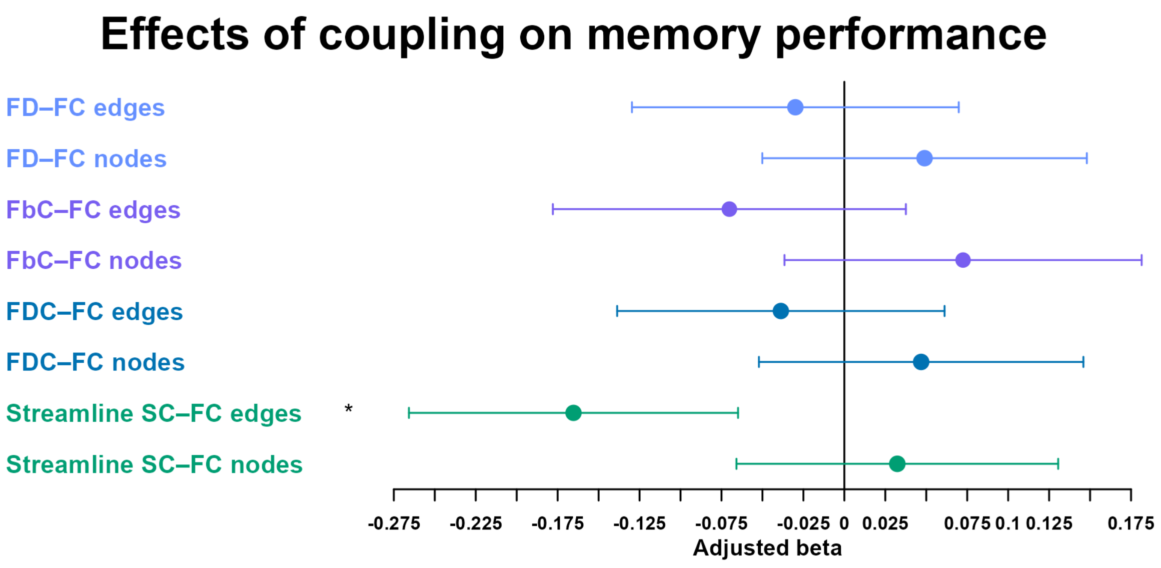

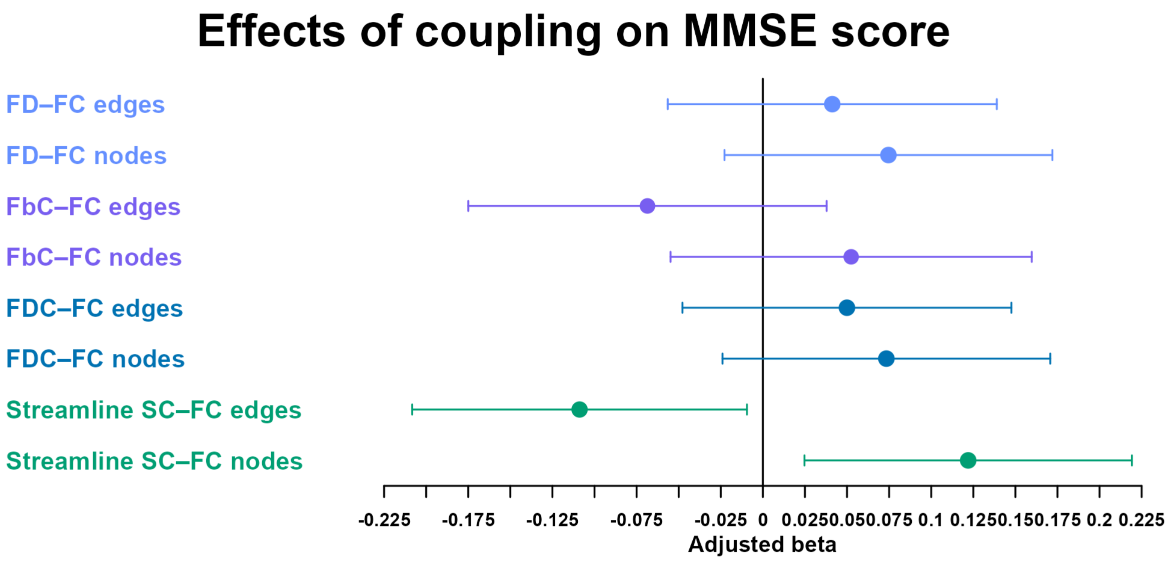

Note. FD=Fibre density. FC=Functional connectivity. FbC=Fibre-bundle cross-section. FDC=Combined fibre density and fibre-bundle cross-section. *significant at *p*=<.05 after correction for false discovery rate. Each bar and dot represent respectively the confidence interval and standardized beta corresponding to the effect of the predictor named on the left. Edge-wise coupling and node-wise coupling were grouped together according to their SC measure (FD,FbC,FDC,streamline) in separate univariate linear models; with age, sex and intracranial volume as control variables. Coupling of "edges" was defined with Pearson *r* correlations between the FC and SC weights of all within-DMN edges in each participant, and coupling of "nodes" was defined with Pearson *r* correlations between the FC and SC nodal strengths of all DMN nodes in each participant.

S4. Coupling analysis with only negative functional connectivity edges

The following results reproduce the same coupling analysis protocol to explore structure-function coupling with *only* negative functional connectivity edges from the FC matrices.

Overall whole-connectome and default mode network coupling

The subject-wise overall coupling, for edges of the whole connectome and of the DMN, across diagnosis groups and SC metrics, are depicted in Figure S7. Diagnosis group had no significant effect on subject-wise whole-connectome coupling for any SC measure (FD: F= 2.607, p=.075; Log(FbC): F= 0.137, p=.872; FDC: F=2.830, p=.060; streamline SC: F=0.603, p=.548). Diagnosis group had no effect on subject-wise overall DMN coupling for any SC measure either (FD: F=0.657, p=.519); Log(FbC): F= 1.267, p=.283; FDC: F= 0.643, p=.526; Streamline SC: F=0.737, p=.479).

Figure S7. Subject-wise overall coupling for the whole connectome and the default mode network across groups and structural connectivity metrics (only negative functional edges)

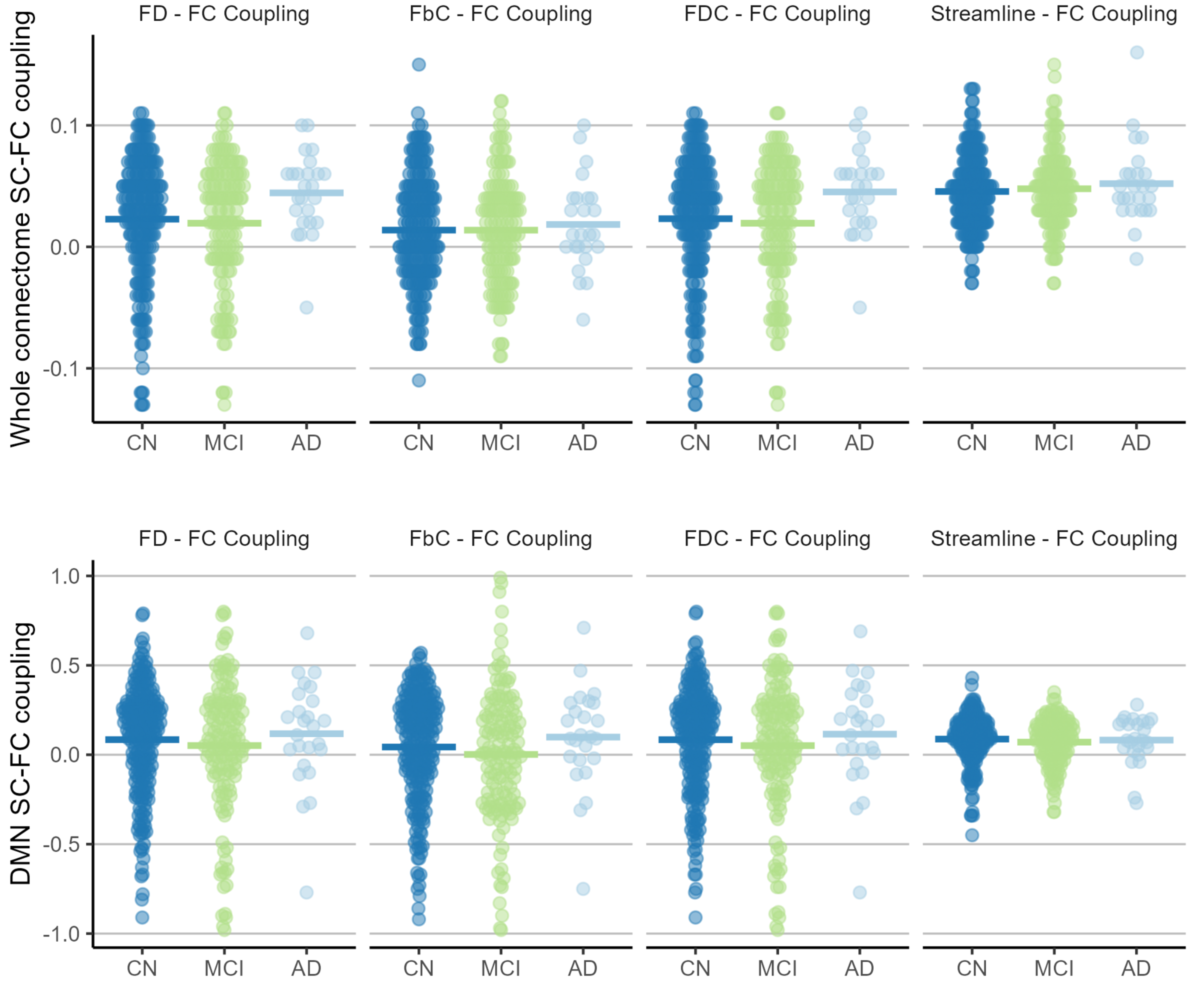

Note. DMN = Default mode network. AD=Alzheimer's disease. MCI=Mild cognitive impairment. CN=Cognitively normal. FD=Fibre density. FC=Functional connectivity. FbC=Fibre-bundle cross-section. FDC=Combined fibre density and fibre-bundle cross-section. Each dot represents a participant's Pearson *r* correlation between the weights of FC and SC edges for the whole connectome (top panel) and for within-DMN edges (bottom panel). The coloured thick bars represent the group mean *r*.

The whole-connectome and within-DMN overall coupling for group averages are depicted in Table S4. No significant difference (Fisher Z transform) in coupling was detected between groups for any SC measure, either for the whole connectome or for connections within the DMN (all $p>.05$).

Table S4 Group-wise average overall coupling for the whole connectome and the default mode network across groups and structural connectivity metrics (only negative functional edges)

	Whole connectome							
	**FD – FC coupling**		**FbC – FC coupling**		**FDC – FC coupling**		**Streamline – FC coupling**	
	r	***p(FDR)***	***r***	***p(FDR)***	***r***	***p(FDR)***	***r***	***p(FDR)***
**AD**	0.122	>.001*	0.122	>.001*	0.124	>.001*	0.171	>.001*
**MCI**	0.103	>.001*	0.103	>.001*	0.106	>.001*	0.219	>.001*
**CN**	0.112	>.001*	0.103	>.001*	0.115	>.001*	0.210	>.001*
	**Default mode network**							
**AD**	0.044	.913	0.011	.941	0.037	.917	0.148	.021*
**MCI**	0.076	.676	0.122	.427	0.080	.677	0.211	>.001*
**CN**	-0.023	.930	0.008	.940	-0.021	.930	0.189	.003*

Note. AD=Alzheimer's disease. MCI=Mild cognitive impairment. CN=Cognitively normal. FD=Fibre density. FC=Functional connectivity. FbC=Fibre-bundle cross-section. FDC=Combined fibre density and fibre-bundle cross-section. * *p* values are statistically significant (p<.05) after correction for false discovery rate.

Edge-wise coupling

The significant edges across SC metrics and groups are depicted in Figure S8.

Figure S8. Edge-wise differences in coupling across groups and structural connectivity metrics (only negative functional edges)

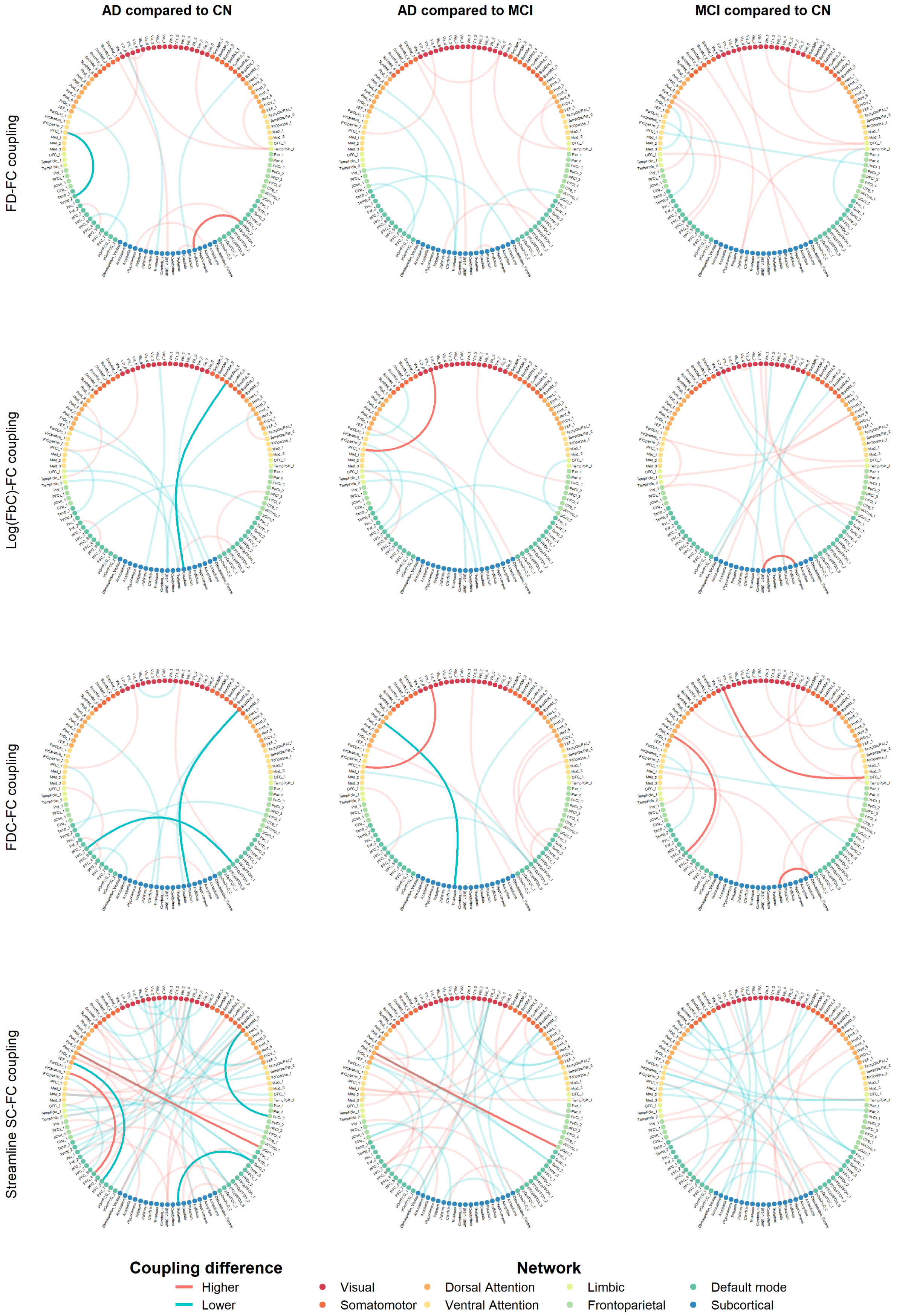

Note. Graphs generated using FCtools R toolbox (https://github.com/CogBrainHealthLab/FCtools/).

Higher = Large *r* value relative to the group AD or MCI is compared to. Lower = Smaller *r* value relative to the group AD or MCI is compared to. AD = Alzheimer's disease. CN=Cognitively normal. MCI=Mild cognitive impairment. FD=Fibre density. FC=Functional connectivity. FbC=Fibre-bundle cross-section. FDC=Combined fibre density and fibre-bundle cross-section. SC=Structural connectivity. Each connecting line represents an edge, where a significant difference between groups in SC-FC coupling was observed. Coupling was defined as the Pearson *r* correlation between the functional weight and structural weight of an edge calculated across the whole group (one SC and one FC weight per participant). Using 5000 permutations, low opacity edges were significant at $p < .0025$ ($p < .01$ with Bonferroni correction for 4 coupling measures), and high opacity edges that survived FDR correction across all edges of all comparisons (corrected $p < .05$).

Node-wise coupling

All nodes where structural and functional node strength coupling significantly differed across groups, for each SC measure, are depicted in Figure S9. No node wise coupling difference was significant after correction for false discovery rate.

Figure S9. Significant node strength coupling differences across groups (only negative functional edges)

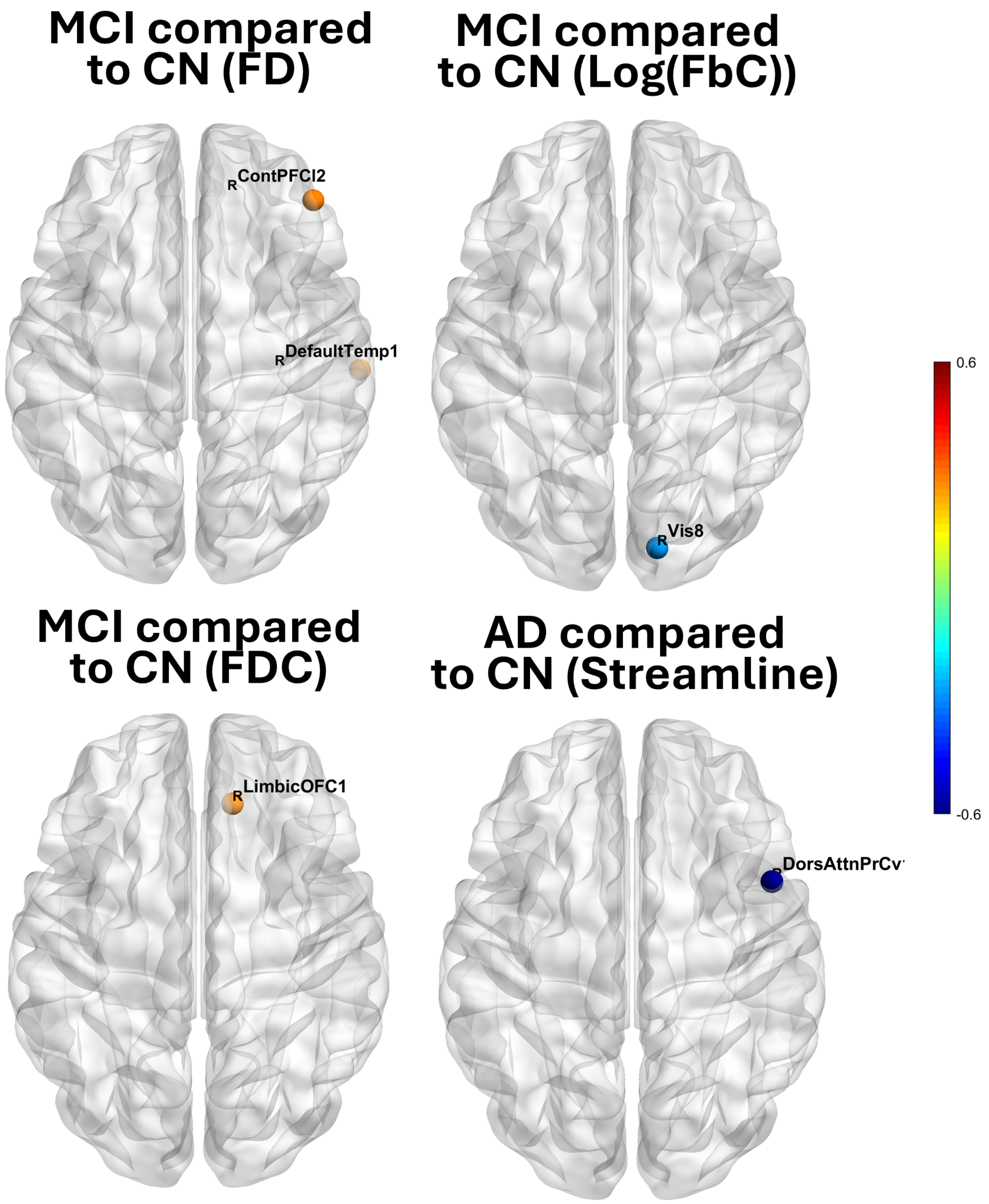

Note. The graphs were produced with the BrainNet Viewer MATLAB toolbox (Xia et al., 2013). Values are raw difference in coupling *r*. AD = Alzheimer's disease. CN=Cognitively normal. MCI=Mild cognitive impairment. FD=Fibre density. FC=Functional connectivity. FbC=Fibre-bundle cross-section. FDC=Combined fibre density and fibre-bundle cross-section. Each coloured dot represents a network node, where a significant difference between groups in SC-FC coupling was observed. Coupling was defined as the Pearson *r* correlation between the functional nodal strength and structural nodal strength

of a node across the whole group (one SC and one FC nodal strength value per participant). Using 5000 permutations, all nodes were significant at $p < .0025$ ($p < .01$ with Bonferroni correction for 4 coupling measures). None survived FDR correction at $p < .05$.

Default Mode Network and cognitive function

Overall coupling of DMN nodal strength did not significantly predict memory performance or MMSE score (Figure S10). Before FDR correction, significant predictors of the memory composite score at p < .05 were FD-FC node-wise coupling in all participants (p=0.016, β=0.122), FDC-FC node-wise coupling in all participants (p=0.010, β=0.130); and there were no significant predictors of the MMSE score.

Figure S10. The effect of default mode network edge-wise and node-wise coupling on memory performance and Mini-Mental Status Examination scores across fixel-based metrics (only negative functional edges)

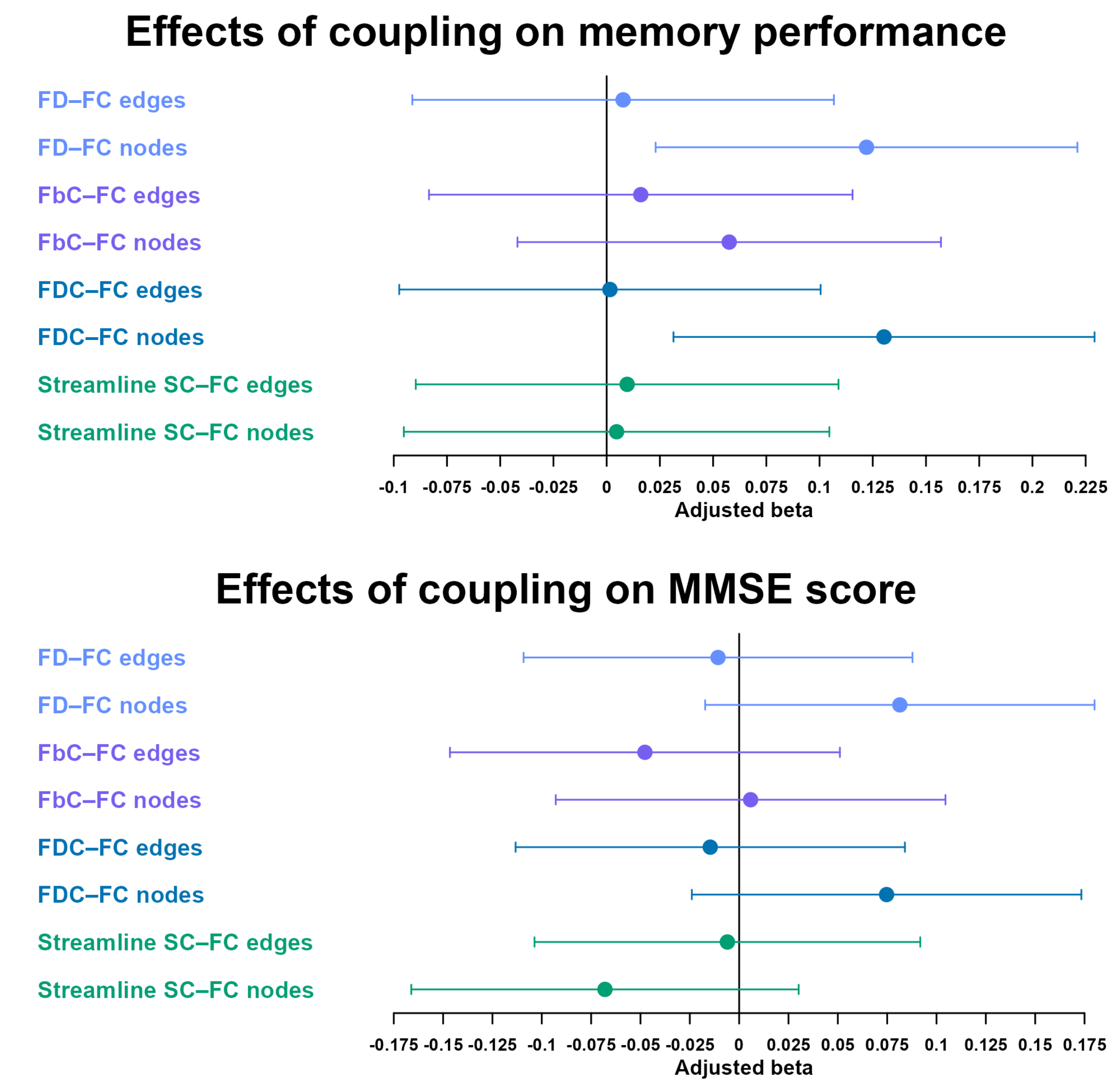

Note. FD=Fibre density. FC=Functional connectivity. FbC=Fibre-bundle cross-section. FDC=Combined fibre density and fibre-bundle cross-section. Each bar and dot represent respectively the confidence interval and standardized beta corresponding to the effect of the predictor named on the left. Edge-wise coupling and node-wise coupling were grouped together according to their SC measure (FD,FbC,FDC,streamline) in separate univariate linear models; with age, sex and intracranial volume as control variables. Coupling of “edges” was defined with Pearson *r* correlations between the FC and SC weights of all within-DMN edges in each participant, and coupling of “nodes” was defined with Pearson *r* correlations between the FC and SC nodal strengths of all DMN nodes in each participant

References:

Xia, M., Wang, J., & He, Y. (2013). BrainNet Viewer: A Network Visualization Tool for Human Brain Connectomics. *PLoS ONE*, *8*(7), e68910. https://doi.org/10.1371/journal.pone.0068910
